# Probiotics, Photobiomodulation, and Disease Management: Controversies and Challenges

**DOI:** 10.3390/ijms22094942

**Published:** 2021-05-06

**Authors:** Laura Marinela Ailioaie, Gerhard Litscher

**Affiliations:** 1 Department of Medical Physics, Alexandru Ioan Cuza University, 11 Carol I Boulevard, 700506 Iasi, Romania; lauraailioaie@yahoo.com; 2 Ultramedical & Laser Clinic, 83 Arcu Street, 700135 Iasi, Romania; 3 Research Unit of Biomedical Engineering in Anesthesia and Intensive Care Medicine, Research Unit for Complementary and Integrative Laser Medicine, and Traditional Chinese Medicine (TCM) Research Center Graz, Medical University of Graz, Auenbruggerplatz 39, 8036 Graz, Austria

**Keywords:** abscopal effect, gut, immune, infections, low-level laser, lung, microbiome, pro-, pre-, syn-, para-, and postbiotics, SARS-CoV-2, COVID-19

## Abstract

In recent decades, researchers around the world have been studying intensively how micro-organisms that are present inside living organisms could affect the main processes of life, namely health and pathological conditions of mind or body. They discovered a relationship between the whole microbial colonization and the initiation and development of different medical disorders. Besides already known probiotics, novel products such as postbiotics and paraprobiotics have been developed in recent years to create new non-viable micro-organisms or bacterial-free extracts, which can provide benefits to the host with additional bioactivity to probiotics, but without the risk of side effects. The best alternatives in the use of probiotics and postbiotics to maintain the health of the intestinal microbiota and to prevent the attachment of pathogens to children and adults are highlighted and discussed as controversies and challenges. Updated knowledge of the molecular and cellular mechanisms involved in the balance between microbiota and immune system for the introspection on the gut–lung–brain axis could reveal the latest benefits and perspectives of applied photobiomics for health. Multiple interconditioning between photobiomodulation (PBM), probiotics, and the human microbiota, their effects on the human body, and their implications for the management of viral infectious diseases is essential. Coupled complex PBM and probiotic interventions can control the microbiome, improve the activity of the immune system, and save the lives of people with immune imbalances. There is an urgent need to seek and develop innovative treatments to successfully interact with the microbiota and the human immune system in the coronavirus crisis. In the near future, photobiomics and metabolomics should be applied innovatively in the SARS-CoV-2 crisis (to study and design new therapies for COVID-19 immediately), to discover how bacteria can help us through adequate energy biostimulation to combat this pandemic, so that we can find the key to the hidden code of communication between RNA viruses, bacteria, and our body.

## 1. Introduction

Naturally, the human body hosts an exceptionally large number of micro-organisms—trillions—which are part of the daily biological life of each individual and support multiple physiological activities with a role in maintaining the integrity and health of tissues, organs, and the whole body.

One of the objectives of this review is to draw attention to the best alternatives in the use of probiotics and postbiotics to maintain the health of the intestinal microbiota and prevent the attachment of pathogens to children and adults.

The second purpose of this review is to update the knowledge about the molecular and cellular mechanisms involved in the balance between microbiota and immune system, for an introspection in the gut–lung–brain axis, to reveal the latest benefits and perspectives of applied photobiomics for health.

The third aim is to reveal and discuss the interconditioning mutual relationships between photobiomodulation (PBM), probiotics, and the human microbiota, their effects on health, and their implications for viral infectious disease management.

The last goal of this review is the urgent need to seek the most innovative treatments to be developed to successfully interact with the microbiota and the human immune system in the coronavirus crisis.

Each human being harbors between 10 and 100 trillion micro-organisms [1] of which the vast majority are in the digestive tract, predominantly in the colon. From 1000 different species of microbes [2], approximately 90% are phylotypes from the Bacteroidetes and the Firmicutes bacteria, which coexist in a symbiotic relationship [1]. These microbes have evolved so that in a healthy specimen they have come to have a mutually beneficial relationship both with each other, and with the host organism. If the organism is in good health, the symbiosis in the microbial community will only bring benefits to both the micro-organisms and the host.

Recent research has discovered new valences in the activity of these micro-organisms that coexist inside our body and on which our well-being depends because they participate in the breakdown of food, help us synthesize vitamins, and protect ourselves against germs that trigger disease. This community of microbes that occupy a well-defined habitat and have distinct physicochemical properties was named microbiome in 1988 by Whipps et al., the term including also “their theater of activity” [3].

## 2. Historical Background

Louis Pasteur (1822–1895) was the first microbiome scientist to make incredible discoveries related to microbial fermentation, pasteurization, vaccination, and to support the theory that microbial germs are the underlying causes of disease [4].

The definition of microbiome comes from the Greek words “micro” and “biom” “Micro” (μικρος) in Greek means small, and the term “biom” originates from the combination of the Greek word bíos (βιος) which means life, to which was added the English suffix “ome”.

A lot of money has been spent in the last decade [5] for research on the human microbiome, which is today recognized as “our last organ” [6]. The accumulation of many scientific materials and more and more knowledge related to the microbiome has produced a paradigm shift in understanding health and disease, and at the same time offers new perspectives for the use of original therapeutical methods based on microbiome control. Although the microbiome is under the influence of a wide variety of stimuli brought by food, physical activity, hormonal secretions, treatments, diseases, it remains almost invariably in the healthy individual [7].

Today, the definition of the microbiome still raises many disputes worldwide, as researchers around the world and various fields of activity have different opinions and have not yet reached a consensus for a unique definition. In the Merriam–Webster Dictionary [8] there are two definitions for the microbiome: one that describes it as a community of micro-organisms (e.g., bacteria, fungi, and viruses) that live in a certain environment and especially that live in or on the human body, where there are approximately 100 trillion bacteria and other microbes [9], and the second one refers to the common genome of micro-organisms living in a particular environment, with reference to the human body [8].

Marchesi et al. [10] claim that the microbiota comprises all living micro-organisms including fungi, algae, and small protists, which should be considered members of the microbiome. They refer to the microbiota as a community of living micro-organisms found present in a well-defined environment.

This definition of the ecological microbiome, based on the environmental aspects of multicellular organisms in a combination of metagenomics, metabolomics, metatranscriptomics, and metaproteomics, does not always correspond to the norms of the microbial world. In the definition of the microbiome, controversies remain mainly related to the integration of micro-organisms such as phages, viruses, plasmids, and genetic elements such as extracellular DNA derived from dead cells (so-called “relic DNA”) [11]. Dupré et al. consider that plasmids, prions, phages, viruses, viroids, and free DNA should not be considered to be living micro-organisms and should not be included in the whole category of microbiota [12].

A definition that is almost generally accepted today is that given by Lederberg and McCray [13], which name the microbiome as a group of micro-organisms within an ecological environment, space, or body, and which live in a close physical association of mutualism or commensalism.

In a recent review of microbiome data, Berg et al. [11] published the results of a recent workshop, which was actively attended by about 40 experts from around the world, as well as the conclusions of an online survey conducted with over one hundred expert researchers in various fields with respect to the study of the microbiome. Summarizing the participants’ responses and those in the online discussions, the scientists concluded that the original definition given by Whipps et al. [3] it is still the most comprehensive, as it combines the complexity of the microbiome with the various aspects of ecology and its biological evolution. During this meeting, the researchers discussed and improved the definition of the microbiome proposed by Whipps et al., and added some recommendations considering the latest developments in current research.

The microbiome combines two defining elements: the microbiota and the “theater of activity”. In this complex, the currently accepted microbiome comprises the microbiota with bacteria, fungi, archaea, algae, protists, together with “theater of activity”, which bring together microbial structural elements (polysaccharides, lipids, proteins/peptides), to which are added structures of DNA/RNA, elements of viral/phage inclusions, relic DNA and microbial metabolites (signaling molecules, toxins, organic/inorganic molecules), etc. [11].

Although the microbiome and the microbiota appear to be synonymous, as shown above, they are separate entities. The microbiome refers to micro-organisms (primary bacteria) and their secondary genes that coexist in a specific environment [14]. The microbiota includes the micro-organisms (microbes) present according to the taxonomy (name), i.e., their type, which live in a specific environment. When we talk about the genes of micro-organisms in an environment, we are referring to the metagenome.

When we refer to ourselves and ask ourselves what role the microbiota has, the answer would be that the human microbiome has an overwhelming influence on health, participating directly in the completion of nutrition, the development of immunity, behavior, and the initiation of the diseases. The human microbiota is labeled as a virtual organ composed of commensal micro-organisms (eubacteria, archaea, filamentous fungi, yeasts, protozoa, viruses) that coexist in symbiosis with our body and have a major impact on digestion, immune system development, cognitive functions, even longevity, as well as maintaining good health [15].

In the body of an adult coexist in symbiosis with the host, trillions of micro-organisms that are spread on the surface of the skin, the mucous membranes of the nasal, oral, vaginal cavities, conjunctiva, saliva, but most of them are in the gastrointestinal tract [16,17]. The structure of the microbiota differs from one individual to another, being specific in direct relation to age, daily diet, lifestyle, ethnicity, environmental factors, etc. [18,19].

The human microbiome begins to form from the intrauterine life with an important colonization at birth by the contribution of the maternal microbiome and other nearby people, as well as by the local composition of the environment [20]. There are studies that show that the intestinal microbiome of infants differs by birth, those born by cesarean section have a lower colonization rate that can persist until the age of about 3 years, when more stable microbial types begin to appear [21,22,23]. The microflora that colonizes the infant’s gut has an important regulatory role for many physiological processes such as nutrient absorption, the development and regulation of the immune system, neurodevelopment, etc. [24,25]. At the same time, a directly proportional relationship was observed between the intestinal microbiome of full-term infants with normal weight, as opposed to a much higher risk of disease (e.g., necrotizing enterocolitis) in children born prematurely or with fetal malnutrition; their intestinal microbiota has an abundance of Proteobacteria and a decrease in Firmicutes and Bacteroidetes [26,27].

Recently, extensive studies on the evolution of the human intestinal microbiota have shown ancestral features of Neanderthal gut microbiome by highlighting the presence of beneficial intestinal commensal bacteria, known as producers of short-chain fatty acids such as *Blautia*, *Dorea*, *Roseburia*, *Ruminococcus*, *Faecalibacterium*, *and Bifidobacterium*. We identify among these, the presence of bacteria that facilitate the extraction of additional energy from dietary fibers, which highlight the importance of plant foods in human evolution, while *Bifidobacterium* provided protective and immunomodulatory benefits to the archaic mother and infant [28]. In addition, the detection of Homo Neanderthal-friendly gut micro-organisms, such as *Spirochaetaceae*, *Prevotella* and *Desulfovibrio*, are now disappearing in Western populations [29], leading to a loss of bacterial diversity in the gut microbiome of the “west”, with parallel growth in autoimmune and inflammatory disorders related to dysbiosis, i.e., “the depletion of health-associated bacteria” [30]. Based on these results, we can already see the new generation of prebiotics and probiotics, as well as other dietary interventions, specific to current individual dysbiosis.

The intestinal microbiota of a healthy individual has a different composition depending on the segments of the digestive tract, and changes throughout life, starting with the infant period and changing with age [31], through the intervention of lifestyle, environment, diversity of food consumed, and by using drugs such as steroids, antibiotics, etc.

Immediately after birth, the microbiota plays the essential role of initiation, training, maturation and functioning of the immune system of the future adult [32]. At the level of the gastrointestinal tract between the microbiota and the host is maintained a balance and a harmonious, beneficial relationship, only when the contact is minimal between the existing micro-organisms and the intestinal surface, which is protected by epithelial cells, mucus, secretory immunoglobulins A (IgAs), immune cells, and antimicrobial peptides, thus limiting inflammatory processes and microbial invasion [33].

Among the millions of micro-organisms in the human digestive tract, there is a class of bacteria that produces molecules and various complex substances, known as probiotics.

The postulate by which the intestinal microflora can be metamorphosed so that harmful microbes can be exchanged with some benefic ones, was issued by the microbiologist Metchnikoff [34].

Élie Metchnikoff (1845–1916) was a Russian-born researcher who worked with Louis Pasteur at the Pasteur Institute in Paris on the study of beneficial microbes and their relationship to health and longevity. He became famous for the results of preliminary research that argued that oral bacteria ingested by mouth could pose a danger of “intestinal self-poisoning”, which would facilitate the aging process [35,36].

Metchnikoff had an extraordinary intuition when he made the connection between the long life of Bulgarian citizens compared to other European peoples, through the daily consumption of fermented dairy products (e.g., yogurt and kefir); so, he is in fact the “founding father of probiotics” [37]. Metchnikoff’s research has drawn particular attention to the ability of *Lactobacillus bulgaricus* to slow down the process of arteriosclerosis and other aspects of aging, which emerge because of the production of uncontrolled intestinal toxins [34,38]. Following published studies on longevity, Metchnikoff is today recognized as the founder of the life extension movement [37,39].

Reports highlighting scientific advice on the evaluation of the safety of probiotics, general guidelines for their evaluation and specific questions related to their pathogenicity, toxicity, and allergenicity, as well as their functional and nutritional properties were preliminary prepared following joint consultations of Food and Agriculture Organization (FAO) of the United Nations and the World Health Organization (WHO), i.e., FAO/WHO experts in 2001, and then developed in the form of a Guide by a group of experts in 2002. Therefore, FAO/WHO experts issued this guide in 2002 defining probiotics as living micro-organisms, which have a positive effect on host health if administered in adequate quantities and established also all the international regulatory statements for probiotics and their safety [40,41].

Commonly bacteria of the genera *Lactobacillus*, *Bifidobacterium*, *Streptococcus*, *Pediococcus*, *Leuconostoc*, *Bacillus*, *and Escherichia coli*, as well as *Saccharomyces* yeast are most often used to modify the microbiota and possibly correct dysbiosis [17]. For example, new types of functional foods could be obtained by inserting probiotics into fruit juices, where they will generate diverse bioactive compounds, with beneficial properties for health from both probiotics and fruit juices [42].

The Human Genome Project, which ran for 13 years (1990–2003), cost approximately $3 billion and succeeded in sequencing the human genome, bringing the greatest benefit in developing an extraordinary and low-cost genome-sequencing technology. When scientists successfully sequenced the human genome, they were amazed to find that the genome has about 23,000 genes, which is substantially less than expected, and even compared to plants, where the number of genes is even double; and the research is ongoing [43].

Other benefits were obtained by funding the next Human Microbiome Project (2007–2016), which published over 350 scientific papers and gave birth to the modern era of microbiome science [44]. Today, scientific and technological advances in the human microbiome allow us to identify compounds generated by various strains of bacteria and understand the health regulatory effects with these products, but at the same time we can identify the most effective bacterial strains in the production of these regulatory compounds.

A remarkably interesting aspect is that the 500–1000 different species of bacteria in our body contain over 3.3 million genes that do not repeat and then it means that over 99% of our body’s DNA is the DNA of our bacteria. This discovery may explain why the human genome contains only 23,000 genes that we can “handle” [37]. Consequently, bacteria use the information contained in the DNA of our body and are directly or indirectly engaged in the release, regulation, and use of the compounds they produce to maintain a healthy microbiome. The results of recent discoveries consider that we are a superorganism controlled by bacteria, so the product of our human genes; that is, we are ourselves plus our bacteria.

## 3. Microbiome and the Immune System

Healthy people accommodate a multifarious group of micro-organisms and other germs living in their gut, bringing multiple and useful support—from helping digestion to the promotion of a healthy immune system. The link between the entire microbial colonization and the initiation and development of various diseases has been studied more and more intensively in recent years, but how probiotics could fight viral infections is of utmost interest in the current COVID-19 pandemic.

The human gut is colonized by an abundant, active, and diverse microbiota [45]. Bacteria, fungi, protozoa, and viruses colonize barrier surfaces of the skin, vaginal, upper respiratory, and gastrointestinal tract, human intestine being populated with as many as 100 trillion cells, whose collective genome, i.e., the microbiome, reflects evolutionary selection not only at host level, but also at microbial cell level [46]. Millions of years of co-evolution have configurated the extraordinary adjustment of the intestinal immune system to maintain homeostasis with a diverse resident microbiota in an incredibly special symbiotic relationship: intestinal bacteria contribute significantly to human nutrient metabolism and instead, live in a nutrient-abundant medium [47].

The signals from commensal bacteria can influence immune cell development and susceptibility to infectious or inflammatory diseases. However, the mechanisms by which commensal bacteria regulate protective immunity after exposure to systemic pathogens remain poorly understood. Experiments have shown that signals from commensal bacteria make operative the innate immune system for an optimal antiviral immunity [48].

However, how can a healthy gut be maintained, and why does the human immune system not attack the 100 trillion beneficial bacteria that populate the gastrointestinal tract, which are foreign, but not harmful?

T-cells emerging from bone marrow progenitors transmigrate to the thymus for maturation, selection, and subsequent export to the periphery are double-trained, once in the thymus, not to attack normal tissues or cells, but to target and eradicate foreign invaders that cause disease and, secondly, after leaving the thymus, in the gastrointestinal tract, so that the activation of regulatory T-cells that inhibit, rather than promote, inflammatory responses to commensal bacteria appears to be a central component of mucosal tolerance [49]. The immune system–microbiota alliance allows the induction of protective responses to pathogens and the support of regulatory pathways involved in the maintenance of tolerance to inoffensive antigens [32,50].

Peripheral T-cells include the following subsets: naïve T-cells (react to new antigens), memory T-cells (maintain long-term immunity after previous antigen activation) and regulatory T (Treg) cells which keep immune responses in check. The roles of T-cells in distinct stages of life evolve from childhood (elimination of the pathogens in infections, improving memory responses and establishing tolerance to harmless foreign antigens), to adulthood (maintaining homeostasis by controlling chronic infections, closely monitoring cancer cells and maintaining adequate immunoregulation) and finally, in old age (reduced function, immunosenescence, cancer and autoimmunity) [51].

Mucosal tissues, such as the intestine and the respiratory track, are continuously attacked by foreign antigens and contain tissue-resident memory T-cells with a superior defensive capacity in antiviral and antitumor immunity [52,53]. As already shown, the immune system evolves throughout the lifespan of humans and undergoes multiple changes in its immunobiology. Last studies have proved that age-related changes in tissues are not necessarily reflected in peripheral blood samples, but of great importance is tissue localization and the delimitation of cellular subsets at different ages [53].

The intestinal epithelium acts as a physical boundary between the microbiota and the rest of the body, senses and responds to microbial signals, and interacts with the vast network of immune cells in and under the intestinal epithelium. The processes involved in the interactions of intestinal epithelial cells–microbe-immunity, however, are not yet fully identified and many unknowns remain with respect to these complex channels of communication [54].

Many intestinal cell types secrete small proteins or cytokines to accelerate cell signaling, activate cell-cell interactions, and control both innate and adaptive immune responses in the gut. These epithelial cells are located between the immune system of the mucosa and the gut microbiome, acting as an arbiter in both directions: intestinal epithelial cells respond to cytokines of immune cells and their response reshapes the microbiome, so through this cytokine signaling network, important functions are tightly controlled such as proliferation, cell death, permeability, microbial interaction, barrier maintenance, keeping the host’s health safe [55].

## 4. Prebiotics, Probiotics, Paraprobiotics, Postbiotics and Synbiotics: Challenges and Controversies

Scientific data attest that the human microbiome has a particularly important role in health and involves its relationship with the emergence of multiple non-communicable diseases [56] but, also infectious, as well as claims that “prebiotics”, “probiotics” and “postbiotics” are considered innovative components of nutrients or foods for good overall health; this information must be disseminated and transposed into health policy [15].

As an increasing amount of scientific data were released concerning the activity of bacteria in our body and, especially, probiotics in the digestive tract in regulating health, researchers began to look for mechanisms of action and explain the relationship with our organs. Are we looking for answers to “how” and “why” probiotic bacteria could adjust our biological activity so well that they prevent and treat a variety of diseases?

The term “probiotic” was adopted in 2001 at an International Meeting of Experts under the auspices of the World Health Organization (WHO) and the Food and Agriculture Organization (FAO) and was subsequently revised in 2014. Definition of “probiotics” includes all micro-organisms that are beneficial to the health of the host when used in the appropriate dose [41], with capacity of survival in the gut without the danger of transferring elements of pathogenicity, antibiotic resistance, and toxicity.

In December 2016, a panel of experts in microbiology, nutrition and clinical research was convened by the International Scientific Association for Probiotics and Prebiotics (ISAPP) to review the definition and scope of prebiotics [57]. All these issues were re-discussed in 2018 in a report by the International Scientific Association of Probiotics and Prebiotics (ISAPP) [58].

Recently, the request of the population for the addition of prebiotics, probiotics [59], and symbiotic in the diet to promote good intestinal health have increased a lot. All those who use these products called “probiotics” should first consult the ISAPP infographic [58]; and a list of “commandments” as has been suggested by Toscano et al. [60].

Presently, probiotics are the subject of comprehensive research to design innovative products, effective marketing, regulation, and rigorous control, and to support consumer interest and safety in prescribing the product by healthcare practitioners. Precisely for these reasons, all these products of the old generation, and especially of the new generation, designed in some cases as living biotherapeutics, must comply with the Nagoya protocol [61]. To achieve this, probiotic strains must have specific characteristics, be safe for the intended use, supported by at least one positive clinical study performed in humans according to generally accepted scientific standards and in sufficient quantity of live product at an effective dose for the entire shelf life [62].

Most probiotic strains represented by the species of lactic acid bacteria, bifidobacteria and yeasts, present on the consumer market are considered to be safe for use in food and as supplements. Brüssow points out that “overstretched negative or positive conclusions from randomized controlled trials with probiotics are to be avoided; the conclusion applies only to the specific probiotic tested against the specified clinical conditions” [63]. Despite all the progress made in recent decades, the mechanisms of action of probiotics are still non-unitary because they depend on the strains in their structure [63].

Microbial strain to be included in the probiotic category must have the ability to adhere to the intestinal mucosa for colonization and modulate the immune system in defense against pathogens [64].

Probiotics can modulate the immune system and have anti-inflammatory activity by the interaction of bacteria with intestinal epithelial cells, dendritic cells (DC), monocytes/macrophages and lymphocytes [65]. Probiotics regulate the host’s immune response by their influence on the innate immune system, as well as adaptive (depends on B and T lymphocytes, which bind to specific antigens).

The innate immune response is obtained through pathogen-associated molecular patterns (PAMP); this response occurs only after pattern recognition (PRR) by PAMP-related receptors. PRRs show TLRs (recognize molecules that are broadly shared by pathogens) that are expressed on immune and non-immune cells, such as B lymphocytes, natural killers, DCs, macrophages, fibroblasts, epithelial, and endothelial cells. PRRs can make connections with lectins, adhesion molecules, and nucleotide oligomerization domains. In addition to TLRs, PRRs also include Nucleotide-like Oligomerization Receptors or NOD-like receptors (NLRs) (also known as nucleotide-binding domain and leucine-rich-repeat-containing proteins), intracellular sensors of PAMP, which protect the cytoplasm space [66].

Currently, there are a lot of scientific attempts to discover the molecular models for the development of anti-inflammatory biomarkers of probiotic bacteria in fermented foods. The improvement of clinical symptoms of some serious diseases such as cancer, diabetes, cardiovascular, metabolic, and allergic disorders, could be regulated by cytokine secretion by intestinal epithelial cells and macrophages under the control of probiotics on various key signaling pathways, such as: NF-kB and mitogen-activated kinases. MicroRNAs, little non-coding RNA molecules, are implicated in transcriptional and post-translational arrangement of gene sequencies by inhibiting the process of genes moving from one place to another.

Effects of the way in which probiotics are influencing the signaling pathways, the pro- and anti-inflammatory activities, and how the cytokines and miRNAs have essential roles in determining the cancerous and inflammatory pathways were investigated in vitro and in vivo in different cell lines and mice models. Studying the match of in vitro and in vivo results, could confirm the correspondence of both modalities, and have a big public health importance in clarifying the role of miRNAs and their signal in inflammation, opening new avenues to pathophysiology, the recognition, and the treatment of different disease in diverse phases of evolution. The results of these studies could lead to the discovery of disease-specific biomarkers for the recognition of the early stages, but also to the study of the influence of different constituents of diets to improve health [67].

Research for functional foods with included probiotics has increased due to many health benefits, such as a strong immune system and anti-inflammatory activity by suppressing pro-inflammatory cytokines (e.g., TNF-α). The mechanisms of action of probiotics in cellular signaling pathways that adjust TNF-α expression are intensively explored [68].

How probiotics really work to suppress all pro-inflammatory cytokines has not yet been fully understood. The comprehensive picture of the exchange of information between probiotics and inflammation-related cellular signaling pathways will help prevent many inflammatory disorders in the future.

Probiotics could play diverse roles through multiple mechanisms on the modulation and stimulation on MAPK (mitogen-activated protein kinase) pathway, on proteasome action, on Toll-like receptors, on NF-kB, especially by inhibiting IkB phosphorylation and reduction, thus hindering the transfer of NF-kB. Effects are strain-dependent, and probiotics of former *Lactobacillus* species play a key role in anti-inflammatory action [68,69].

The differences between “paraprobiotics” and “postbiotics” are that paraprobiotics are considered lifeless or inoperative probiotic cells, while “postbiotics” are tonic metabolites of probiotics, both with common origins that are widely studied in functional biotics. Postbiotics have multiple benefits over conventional probiotics, through the molecular structure, which is already known, are used in purified compounds, have a specific activity, and intervene more easily on microbe-associated molecular pattern (MAMP), i.e., on MAMP-PRR to promote the downstream path. Another difference is that they are easy to make industrially through easy processes of production, packaging, transport, storage, administration etc. [70].

All categories of probiotics have anti-inflammatory activities, act as intestinal barrier against pathogens, are anti-adhesion to harmful micro-organisms, anti-biofilm, antivirals, modulators of the immune system, reduce blood pressure and cholesterol, are antiproliferative, apoptotic and anti-oxidant and so on (Table 1 and Table 2).

Bifidobacteria and lactic acid strains have the largest coverage area with probiotic properties and are integrated into many functional foods and dietary supplements. The beneficial effects of probiotics are expressed by their ability to prevent and relieve various symptoms such as: acute diarrhea secondary to antibiotic therapy, allergic manifestations (eczema, allergic rhinitis, conjunctivitis, wheezing), diarrhea with *Clostridioides difficile*, inflammatory bowel disease, type 2 diabetes, etc. (see Table 2—Clinical applications)

There is the following classification: (a) probiotics (fermented foods); (b) foods with a Generally Recognized As Safe (GRAS) status, such as *Lactobacillus*, *Bifidobacterium and Lactococcus*; (c) dietary supplements, sold as over-the-counter (OTC) supplements; and (d) medicines (pharmaceuticals) [42].

The potential of probiotics and postbiotics in participating in changing the physiological state of the host, to prevent the disease or improve its condition, is recognized by studies published so far. For example, *Lactobacillus rhamnosus GG* (LGG) and *Bifidobacterium animalis* subsp. *lactis* BB-12, can bring great intestinal benefits, but the effect was not always observed in decreasing the total number of picornavirus in different protocols, so that extra studies are necessary in elucidating the peculiar antiviral action of these two probiotics against rhinoviruses [69]. Multiple clinical trials in humans, and double-blind randomized and placebo-controlled studies are still needed to confirm the bioactive properties of these probiotics. Moreover, additional investigations are required on immunocompromised patients because they have a higher risk of adverse reactions. There are uncertainties regarding the stability, bioavailability, and interaction with ligands in the digestive tract, to know more precisely the mechanisms of action both in vitro and in vivo [70] (Table 1).

Research has shown that even non-viable micro-organisms could be helpful and triggered the application of non-viable probiotic preparations, known as “paraprobiotics”. Many disadvantages associated with the administration of viable micro-organisms, i.e., the lack of stability under certain storage conditions, are eliminated. Paraprobiotics could substantially decrease the functionality problems and remove the risks of microbial translocation and consumer infections, promising natural antibiotics alternatives. Paraprobiotics provide health benefits by adjusting the immune system, increasing the adhesion to gut cells by inhibiting pathogens, and different metabolites are contained [70,79,87,100,101].

Globally, there are special concerns for children’s health and there are still high infant mortality rates, which are also extended until the age of five, especially in countries with a low standard of living. Respiratory and digestive tract infections are a major public health problem, especially for preschoolers [105].

Programs developed by the WHO and other international organizations on the education, care, and protection of mothers and children have reduced the infant mortality rate since the 1990s, but the level remains high for newborns and children under five [118].

If we try to make an analysis of age and causes of death, we see that the highest death rates are for newborns and then they gradually decrease to the age of five; the main causes are age and weight at birth, the mode of birth, genetic factors, diet, and infectious complications, especially severe digestive disorders (e.g., necrotizing enterocolitis) [119]. Microbial agents involved come from the bacterial species *Shigella*, *Salmonella*, *E. coli*, *Yersinia enterocolitica*, *Campylobacter jejuni*, *and* entero-invasive viruses such as *Rotavirus*, which sometimes cause very severe forms of disease with a high mortality rate, especially in children with a low standard of living [120].

Protecting the infant and young child from serious digestive infections can be achieved by developing a healthy microbiome that participates in the initiation and strengthening of a strong immune system. In the first 6 months of life, breastfeeding would play an essential role in developing a healthy immune system and protecting the baby from illness. However, there are many reasons why the baby cannot be breastfed, and in this sense, there have been many humanized powdered milk formulas (structures close to breast milk) and even improved with probiotics. The latest are microbial agents with amazingly complex functions, because through their metabolic activities they manage to digest and ferment fiber from food, which promotes the release of a wide variety of compounds that regulate health, and they are called “postbiotic metabolites” [121,122].

Probiotics, prebiotics and synbiotics are increasingly used today with valuable results as growth promoters and alternatively as prevention products against several enteric pathogens [100]. Postbiotics are a substrate used selectively by host micro-organisms, which confer health benefits [123].

Administration of probiotic bacteria as a food adjunct in health promotion has a long and successful history without side effects, for which they have received the GRAS status.

However, in some cases, probiotics of the genera *Lactobacillus*, *Leuconostoc*, *Pediococcus*, *Enterococcus* and *Bifidobacterium* have been suspected of triggering infections in immunocompromised patients [124]. Probiotics, through surface proteins act competitively in the intestinal lumen fighting with pathogens for adhesion to mucus or intestinal cells and thus manage to block and prevent the invasion of the intestinal wall [125]. Consumption in large quantities of one or more strains of probiotic bacteria can have negative effects on the immune system. Wen et al. reported that “probiotics can be ineffective or even harmful if not used at optimal doses” [126].

Regarding the use of probiotics, there are several reasons for concern due to side effects [127], such as: bacteremia, necrotizing enterocolitis, pneumonia, and meningitis [128]. To date, publications on the side effects of probiotics show that they are generally safe [129], but there are some studies that have shown that probiotics can be theoretically responsible for four types of risks, as follows: systemic infections, harmful metabolic activities, excessive immune stimulation in susceptible individuals, and gene transfer (Table 3).

As a conclusion for Table 3, the main side effects of probiotics are related to bacteremia/fungemia, with predilection found in premature newborns, elderly, immunosuppressed or critically ill patients with severe or fatal comorbidities, or patients in intensive care units treated with broad-spectrum antibiotics on central venous catheters.

Data from the literature support the great potential of the application of probiotics in many pathologies and especially in those recently induced by RNA viruses, such as SARS-CoV-2. However, there are many publications that warn that probiotics should be used with caution, especially in people with non-communicable diseases. At the same time, special care should be taken when using probiotics in the elderly and especially in immunocompromised or heart disease patients, corrected by valve prosthetic implants, for which there is a risk of infection and transmission of resistance genes, in especially in patients with prolonged antibiotic therapy. A better knowledge of the mechanisms of action and of the biochemical profile could open new applications in the prevention and therapy of COVID-19 [151].

Another concern comes from the fact that some strains of probiotics could express virulence factors, which increase the ability to adhere, invade and trigger cytotoxicity [152]; moreover, they could collect from the environment into their genome antibiotic-resistant genes that can then be transplanted to other pathogenic bacteria in the digestive tract [153,154,155,156].

Of all the known categories, the safest appear to be postbiotics for which no major adverse reactions have been yet reported. Use of postbiotics has been proposed as an alternative to probiotics, to help reduce the incidence of infectious diseases in infants and preschool children.

Along with user awareness, to optimize the positive effects of probiotics on human health, food products containing postbiotic compounds have been introduced [157]. Postbiotics are biotherapeutic products derived from inactivated probiotic strains, or their metabolic products, or both, following a fermentation process and which are used to maintain the integrity of the intestinal barrier and promote the health of patients at high risk of disease [158]. Postbiotics are beneficial in terms of safety, biological properties, absorption, transport, metabolism, distribution, excretion, proper signaling to various host organs and tissues, and for pharmaceuticals, as they do not include any risk of translocation from the intestinal lumen into the blood, compared to the living probiotics [75]. Postbiotics strengthen host endogenous probiotics in the intestinal microbial ecosystem [159,160,161] to prevent disease, strengthen the immune system and as complementary therapeutic alternatives [157].

Paraprobiotics and postbiotics as probiotic derivatives are used today in humans, animals, and birds for their immunostimulatory, anti-inflammatory, anti-oxidant, antimicrobial properties, as well as for growth promoters [75]. Postbiotics are currently available in some infant formulas and fermented foods.

Recent studies point out that postbiotics can become alternative agents to probiotics that contain living micro-organisms and can be used in the fields of human medicine, veterinary medicine, and the food industry to prevent and treat diseases, or to support animal health and functional food [162]. Postbiotics and paraprobiotics have a valuable opportunity for their expansion as functional biotechnological products for the nutraceutical industry [74].

In his book The Mind-Gut Connection, the author Emeran Mayer states that “postbiotics” or “postbiotic metabolites” produced by bacterial strains have a role in reducing inflammation, regulating the acid-base balance inside the digestive tract, direct inactivation of pathogens, regulating the process of digestion, absorption of nutrients, detoxification, regulation of the immune system and the permanent transmission of information from the intestine to the brain [163].

If we consider that the bacteria in our body will produce “hundreds of thousands of metabolites” with a particularly important role in maintaining perfect health, it is imperative that postbiotics now become the new frontier in microbiome research. Some of the postbiotic metabolites are glutathione synthesized by *Lactobacillus fermentum* ME-3 [164], B vitamins (biotin, cobalamin, folate, nicotinic acid, pantothenic acid, pyridoxine, riboflavin and thiamine) and K vitamin [165], antimicrobial peptides (AMP) [166], D-amino acids [167], hydrogen peroxide [168] etc. Postbiotic metabolites send millions of biochemical signals, which play a defining role in the functioning of the biotope, regulate the health of the body, and have become a new frontier of microbiome science.

As is known, probiotic bacteria in the gut need time to locate and digest dietary fiber to release postbiotic metabolites, so in the case of symptoms related to dysbiosis, the best way is to ingest the postbiotic product orally. After ingestion of postbiotic metabolites resulting from the fermentation process, it immediately enters the health promotion action, by decreasing the inflammatory process, balancing the acid-base balance, stimulating the division and development of healthy cells that attenuate the intestinal wall, destroying abnormal pathogenic micro-organisms, and restoring the connection between the gut and the brain [169]. Mechanisms by which postbiotics work and their involvement in maintaining the health of the host are not fully known. Research results show that inactivated probiotics or their components can adjust the host’s immune system through bacterial film, capsule, or peptidoglycan structures, liposaccharides [170] and S-layer proteins of the cell wall [171,172].

We are currently discussing the participation of postbiotics through two mechanisms, one is the involvement of the innate immune response and the second is the acquired immune response, which consists of recognizing receptors [173] with the ability to associate with micro-organisms [174].

At the level of host cells there are two receptors for the recognition of bacterial metabolites: receptors in the field of nucleotide-binding and oligomerization (NOD)-like receptors (NLRs) and the toll-like receptors (TLRs) [175].

NLRs can recognize several ligands from microbial pathogens, including peptidoglycans, flagellin, viral RNA, etc. [176]. After activating NLRP1, they form a multiprotein structure called inflammasome-NLRP1 which is exposed on macrophages, T lymphocytes, epithelial cells, dendritic cells (DC), innate and adaptive immune cells [177]. To respond to postbiotic stimuli, NLRs may respond to various cytokines, including interferon, and participate in the activation of T and B lymphocytes [178]. In this way, there is the possibility of activating caspase-1, which will promote the release of pro-inflammatory cytokines, interleukin 1 (IL-1) and IL-18 [179].

TLRs are a family (TLR 1–8) of receptors capable on the one hand to recognize pathogens, and on the other hand after being activated to bind to a bacterial component and activate the immune cells that will produce a certain type of cytokine (signaling molecule) [180]. Interestingly, TLR9 located on the basolateral side of the intestinal epithelial cell membrane activates a remarkably interesting field, namely the kappa B nuclear factor (NF-kB) pathway with a role in the release of pro-inflammatory cytokines; and, if it is found in the apical area, it plays an inhibitory role [181].

Cytokines may have pro-inflammatory or anti-inflammatory properties; to avoid an exaggerated inflammatory response or immunosuppression, there must be a balance between these two types of signaling molecules. Results of human clinical studies have shown beneficial effects between the consumption of fermented foods containing postbiotic metabolites such as short-chain fatty acids (SCFAs), like: acetic, propionic, and butyric acid, used to treat diseases, for example obesity [182,183], type 2 diabetes [184], depression [185], hyperlipidemia [186], osteoporosis [187], malnutrition management [188], infectious diseases common in children [189,190,191], and recently SARS-Cov-2 infections [192,193].

Children under the age of five are extremely vulnerable to infections because the dowry of protective factors inherited from the mother is lost during aging and, on the other hand due to the immaturity of the immune system [194].

Malagón-Rojas et al. published a systematic review of randomized clinical trials to highlight evidence of the benefits of using postbiotics in the prevention and treatment of common infectious diseases among children under 5 years of age. The authors point out that there are not enough randomized studies; however, postbiotics could be a suitable alternative for the treatment of diarrhea and the prevention of the frequency of infectious diseases in children [105]. The authors studied the activity of three probiotic strains of *Lactobacillus* (or a postbiotic) and compared it with the pathogenic *Salmonella* strain in the culture of healthy intestinal mucosa and inflammatory bowel disease (IBD). The study shows that probiotics are not always beneficial to the healthy host and can also be harmful in active IBD, while a valuable postbiotic can protect against the aggressive inflammatory activities of invasive *Salmonella* types, and at the same time could regulate the inflammatory processes present in the tissue with IBD [195].

A recent study published by Nataraj et al. claim that postbiotics are a complex of metabolic products secreted by probiotics in cell-free supernatants, consisting of enzymes, proteins, short-chain fatty acids, vitamins, secreted biosurfactants, amino acids, peptides, organic acids, etc. Paraprobiotics are inactivated or broken microbial cells that contain peptidoglycans, teichoic acids, surface proteins, or extracts of crude probiotic cells. Postbiotics and paraprobiotics have many more advantages over probiotics through availability in pure form, lightness in industrial production and storage, the specific mechanism of action and an easier approach in recognizing and interacting with host receptors [70]. Research conducted and published to date claims that postbiotics can act by direct immunomodulation and there is clinically evidence for the effect of improving general health and symptoms of abdominal pain in adults, childhood colic, atopic dermatitis and various etiologies of diarrhea [196].

It has recently been shown that *Candida auris* (*C. auris*), by its ability to produce biofilms, eludes the immune capacity of the host and antimicrobial agents, becoming an important pathogen with remarkable resistance to antifungal agents. Rossoni et al. studied the antifungal action, using in vitro and in vivo models of the probiotic cells *Lactobacillus paracasei* 28.4 and the postbiotic activity of the crude extract (LPCE), as well as fraction 1 (LPF1), from the supernatant *L. paracasei* 28.4. The results showed that after 24 h of treatment with LPCE or LPF1 there was a complete reduction of viable *C. auris* cells compared to fluconazole, significantly reduced biomass (*p* = 0.0001) and metabolic activity (*p* = 0.0001) of *C. auris* biofilm and a total reduction of *C. auris* cell viability persists after treatment with postbiotic elements (*p* < 0.0001) [197].

Disorders caused by premature colonization of the digestive tract, in combination with the immaturity of intestinal barrier defense factors and the aggressiveness of mucosal colonizing bacteria are directly involved in the pathogenesis of necrotizing enterocolitis [198]. Recent advances in understanding the mechanisms of action and biological effects of postbiotics have recommended their use as an effective and promising preventive measure against necrotizing enterocolitis, removing the risks of using live micro-organisms in premature infants and infants that could translocate and cause infections [199].

Lactobacilli are widely used as probiotics with beneficial effects on infectious diarrhea, necrotizing enterocolitis, and IBD. However, in patients with a disturbed intestinal epithelial barrier, it is preferable to use metabolic products called postbiotics, as they could prevent possible side effects caused by live bacteria.

Haileselassie et al. studied in vitro how *Lactobacillus reuteri* DSM 17938 cell-free supernatant (*L. reuteri*-CFS) influenced mucosal-like retinoic acid (RA) derived from dendritic cells (DC) and the effect on regulatory T lymphocytes (Treg). RA generated a mucosal-like DC phenotype with elevated levels of IL10, CD103, and CD1d and a decrease in mRNA expression from several inflammation-associated genes (NF-κB, RelB, and TNF). In conclusion, *L. reuteri*-CFS modulated the mucosa and DC function, as a biologically active molecule in the phenotype of the supernatant, proving its potential activity as a postbiotic [103].

Postbiotics today provide a halo image due to their clear chemical structure, safety doses, prolonged expiration date and complex structure with signaling molecules that can have immunomodulatory, anti-inflammatory, anti-oxidant, antiproliferative, anti-obesity, antihypertensive, and hypocholesterolemic activities. All these qualities recommend postbiotics for administration to improve specific physiological functions and host health, even if the mechanisms of action have not yet been fully elucidated [75].

In an in vitro study, Aguilar-Toalá et al. investigated the multifunctional bioactivities of intracellular content (IC) and cell wall fractions (CW) obtained from *Lactobacillus casei* CRL 431 and *Bacillus coagulans* GBI-30 strains. Several compounds (fatty acids, amino acids, coenzyme, proteins, amino acids) with probable significant activities (Pa > 0.7) were highlighted as immune-stimulating, anti-inflammatory, neuroprotective, antiproliferative, immunomodulatory, and antineoplastic. In vitro tests demonstrated that the IC and CW fractions showed inhibitory activities of the angiotensin converting enzyme (>90%), chelating agents (>79%) and antioxidants. The results based on in silico and in vitro analyzes suggest that *L. casei* CRL 431 and *B. coagulans* GBI-30 strains appear to be promising sources of postbiotics and may confer health benefits through their multifunctional properties [200].

Heat stress is a major problem in poultry farms in hot and humid countries because it affects their health and productivity. The widespread use of antibiotics to reduce stress and infectious diseases and as growth promoters has led over time to the emergence of antibiotic-resistant bacteria and the possibility of antibiotic-resistant genes to be transferred between birds.

Postbiotics produced by *Lactobacillus plantarum* have begun to be studied extensively as an additive to replace antibiotics in animal feed, but no studies have investigated the role of postbiotics in feed for chickens under heat stress [201].

Humam et al. estimated the effects of different postbiotic administration on carcass growth yield, intestinal morphology, microbiota, immune status, growth hormone receptor (GHR) and insulin growth factor 1 (IGF-1) gene expression in chickens under heat stress. A total of 252 chickens randomly distributed in identical environmentally controlled cages were studied, divided into 6 groups. Results show that postbiotic supplementation of chickens subjected to heat stress significantly improved the height of the duodenum, jejunum, ileum, and the depth of the duodenum and ileum crypts, compared to those treated with the basal diet. The postbiotic RI11 group recorded a significantly higher number of *Lactobacillus* bacteria in caecum, with a lower number of *Salmonella* compared to the basal diet groups; At the same time, an increase in hepatic expression of GHR mRNA, hepatic IGF-1 mRNA, and plasma levels of immunoglobulin A, M, and G was observed compared to the control group. The study proved that *Lactobacillus plantarum* could be used as an alternative to antibiotics, as a growth promoter and anti-infective and anti-stress treatment in poultry farms [201].

Mechanisms by which resident microbial species impact on gastrointestinal pathogens are complex and include competitive metabolic interactions and the production of antimicrobial molecules. Certain probiotics secrete molecules with antibiotic-like activities, playing important roles in cell regulation, as well as with significant therapeutic effects proven by clinical research. These anti-infective molecules called lantibiotics are a promising new source for the development of innovative anti-infective agents that act luminal and intracellularly in the gastrointestinal tract, important for their use in the case of infections (i.e., antibiotics) [202].

Simultaneously with the reduction of antibiotics in poultry feed, an extremely dangerous pathology appeared with a high mortality, ulcer-necrotic enteritis. The discovery of alternative products to antibiotics has become extremely urgent. Postbiotics, as non-viable bacterial products or metabolic byproducts from probiotic micro-organisms, have positive effects on the intestinal microbiota and are a promising alternative to antibiotics [203].

## 5. Probiotics in the Management of Various Pathologies: Perspectives in COVID-19

### 5.1. Probiotics in Digestive Tract Pathology

Diarrhea secondary to prolonged administration of antibiotics is a common side effect caused by an imbalance of the intestinal microbiota. The most common pathogen is *Clostridioides difficile*, which through resistance to antibiotics causes infection of the large intestine [204].

Vanderhoof et al. studied the efficacy of *Lactobacillus casei* subsp. *rhamnosus* (*Lactobacillus GG*) (LGG) in reducing the incidence of antibiotic-associated diarrhea when co-administered with an oral antibiotic in children with acute infectious disorders. The study was done randomized double-blindly on 25 children with diarrheal disease; in the end, LGG reduced the incidence of antibiotic-associated diarrhea in children treated with oral antibiotics for common childhood infections [205].

Antibiotics can cause a microbial imbalance in the gut resulting in antibiotic-associated diarrhea (AAD). Probiotics can prevent AAD by rebalancing the intestinal microflora, repairing the intestinal barrier, etc.

Probiotics are increasingly used to prevent and treat diarrheal disease more in children than in adults.

Guarino et al. undertook research on randomized controlled trials of digestive pathology that included: acute gastroenteritis, antibiotic-associated diarrhea (AAD), and necrotic enterocolitis (NE) [206]. In acute gastroenteritis he found 12 studies: 5 with recommended probiotics and 7 not. LGG and *Saccharomyces boulardii* had the most convincing evidence of efficacy, as they reduced the duration of the disease by one day. For AAD, 4 meta-analyzes were found, which show the variable efficacy of probiotics in preventing diarrhea, depending on the patient’s age and the antibiotic used. The most effective strains were LGG and *S. boulardii*. In the case of NE, 12 studies were analyzed (of which 3 were randomized controlled trials) and it was found that probiotics reduced the risk of NE and mortality in premature infants. The guidelines did not support routine use of probiotics and requested additional data for such sensitive implications. Research proved there is strong and solid evidence of the effectiveness of probiotics as an active treatment of gastroenteritis in addition to rehydration. There is strong evidence that probiotics have some effectiveness in preventing AAD, but the exact dose needed for treatment is a problem. For both etiologies LGG and *S. boulardii* have the strongest evidence. In the NE, indications are more debated, but based on available data and their implications, probiotics should be considered carefully. One of the most common side effects during antibiotics is diarrhea. Probiotics are living micro-organisms that, after oral ingestion, can prevent antibiotic-associated diarrhea by normalizing the unbalanced gastrointestinal flora [206].

A meta-analysis was performed by Blaabjerg et al. [204] on the benefits and side effects of probiotics used to prevent AAD in an outpatient setting. A search of the PubMed database was performed and a total of 3631 subjects were included in the analysis. The cumulative results found that 8.0% of the probiotic group with LGG and *S. boulardii* strains had AAD, compared to 17.7% in the control group. No statistically significant differences were demonstrated in terms of the incidence of side events. The results suggest that the use of probiotics may be good and safe in preventing AAD.

Guo et al. evaluated the efficacy and safety of probiotics used to prevent AAD in children. Thirty-three studies were included (6352 participants) by search: MEDLINE, Embase, CENTRAL, CINAHL, and Web of Science (as of 28 May 2018), including ISRCTN and clinicaltrials.gov. The probiotics evaluated included *Bacillus* spp., *Bifidobacterium* spp., *Clostridium butyricum*, *Lactobacilli* spp., *Lactococcus* spp. *Leuconostoc cremoris*, *Saccharomyces* spp. or *Treptococcus* spp., alone or in combination. The results suggest a moderate protective effect of probiotics for the prevention of AAD. Using five criteria to assess the credibility of the probiotic dose subgroup analysis, the results indicated that the effect of the high-dose probiotic subgroup of over 5 billion colony-forming units (CFUs) per day was credible. Evidence also suggests that probiotics may moderately reduce the duration of diarrhea, a reduction of almost a day. The benefit of high-dose probiotics (e.g., LGG or *Saccharomyces boulardii*) should be confirmed by a well-designed randomized multicenter study. Adverse event rates were low, and no serious side effects were attributed to probiotics [207].

Analyzing the effect of AAD probiotics concomitantly with the use of antibiotics, Yan et al. showed that two probiotics (LGG and *S. boulardii*) are effective in preventing pediatric AAD when administered concomitantly with antibiotics. The optimal dose remains unknown, but 5 to 40 billion CFUs per day seems to be the most effective. These appear to be safe in children, with minimal side effects; however, serious adverse events have been documented if the children were severely debilitated or immunocompromised [208].

### 5.2. Probiotics in Pulmonary Viral Infections

Discovery of the human genome and recent innovative high-speed and low-cost sequencing technologies of genes, especially the 16S rRNA gene [209] disturbed the conservative idea that the lung would be sterile.

The concept of lung sterility [210] was supported by laboratory data limited by traditional study techniques by aspiration of secretions and then their culture, which detected a percentage of only 1% of bacteria present in healthy airway samples [209].

Progressive-minded ideas and the accumulation of a huge number of studies on the microbiota in the last decade have reformed our understanding of the existence of the lung microbiota and the lung–microbiota axis (relationship) [211].

More and more studies provide evidence of the strong relationship between the intestinal microbiota and many human diseases [6], and the recognition in depth of the dual host-microbe interaction mechanisms in the intestine and lung is a necessity, to be able to prevent, detect and apply in diseases therapy [212].

The pulmonary microbiota plays a particularly important role in preserving the homeostasis of the respiratory system, to promote and preserve a state of immune tolerance, to prevent an unwanted inflammatory reaction after inhalation of harmless environmental agents. This activity is supported by an indestructible and permanent link between the microbiota and the immune cells in the lungs, which through specialized sensors detect invasive micro-organisms [213].

The oropharynx and the upper respiratory tract are permanently invaded by microbes that through direct communication and subclinical aspiration of the oropharyngeal content, enter the lungs and form the bacterial microbiome in various anatomical sites.

Changes in the lung microbiome through which dysbiosis can occur, will influence the host’s immunity and defense; understanding these complex interactions between the host and the pathogen elucidates the pathogenesis of chronic lung disease [214].

Once the respiratory tract infection has occurred, the commensal microbial flora acts locally on the lungs and on the intestine-lung axis and an adjacent immune response occurs [212].

Laboratory research on murine has shown that the bacterial flora in the lungs grows immediately after birth, so at 15 days we find fewer strands of Gammaproteobacteria and Firmicutes, and many more Bacteroidetes [215].

During the development and growth of the infant and later the child, the lung is increasingly populated with various bacteria, up to the mature microbiota.

Experimental studies have shown that between the intestinal microbiota and the segments of the respiratory system there is an interconnected relationship, for example: disruption of the intestinal microbiota in mice by antibiotics led to increases in fungal colonies, which exaggerated the immune response (increased eosinophils, mast cells, serum levels of IL-5, IL-13, IFN-γ, IgE) allergic to intranasal provocation with *Aspergillus fumigatus* [216].

Administration of probiotics for the modulation of the intestinal microbiota in *Macaque* monkeys led to an increase in the number of B lymphocytes expressing IgAs in the colon and in the lymph nodes, probably as a response to the growth of T-helper follicular cells (Tfh) and IL-23 expression in dendritic cells [217].

Acute infections of the upper respiratory tract and lung of viral etiology (adenovirus, rhinovirus, influenza, enterovirus, coronavirus) then complicated bacterially, is a major public health problem worldwide, a major cause of debility, chronicity and death in children and adults [218].

RNA viral agents are known to be extremely contagious and can cause respiratory infections such as Severe Acute Respiratory Syndrome (SARS) and even a pandemic, such as the current “Coronavirus disease 2019” (COVID-19), a contagious infection produced by severe acute respiratory syndrome coronavirus 2 (SARS-CoV-2) [219].

In this pathology, the best attitude is to prevent viral infections knowing that antiviral drugs are few, and vaccines are limited.

Probiotics can be a valuable alternative for preventing and ameliorating respiratory tract infections with viral agents, which cause so many diseases in children and adults.

Maeda et al. studied the effect of the oral *Lactobacillus plantarum* L-137 (HK-LP) probiotic in mice infected with intranasal administration of influenza A/FM/1/47 virus (H1N1, a mouse-adapted strain). They found that clinically, survival time was prolonged in the probiotic group and that viral titers were significantly lower than in the control group. Biologically, an elevated level of interferon beta (IFN-β) was demonstrated in HK-LP-treated mice, while in the control group it was undetectable. The authors concluded that the probiotic HK-LP was beneficial in preventing the spread of influenza infection by inducing IFN-β synthesis [220].

Several in vitro and in vivo studies in mice have shown that HK-LP, an isolated strain of fermented food, was a potent stimulant for the synthesis of cytokines IL-12 and tumor necrosis factor alpha (TNF) -α) [102,221,222,223].

Hori et al. [224] demonstrated that intranasal administration of *Lactobacillus casei* strain *Shirota* (LcS), produces a strong release of IL-12, interferon-gamma (IFN-γ) and TNF-α, which have an important effect in eliminating influenza virus from mediastinal ganglion cells. Reducing the virus titer in the upper respiratory tract to 1/10 compared to the control group was valuable in preventing the death of the studied mice. This study suggests that intranasal administration of LcS improves the level of cellular immunity in the respiratory tract and prevents infection with influenza virus.

Lehtoranta et al. [225] conducted a review of the effects of probiotics administration (*Lactobacillus*, *Bifidobacterium*, *Lactococcus*) on viral respiratory tract infections in animal models and clinical trials, and found promising data demonstrating that specific probiotics can shorten the duration or reduce the risk of respiratory infections.

Arshad et al. [226] in a recently published mini review, show that the use of plant-based foods in the daily diet with high levels of minerals such as magnesium, zinc, micronutrients, vitamins C, D, and E, along with a good lifestyle, increase the number of good intestinal bacteria that boost the immune system and can control the onset of respiratory viral infections, including COVID-19.

Pulmonary microbiota, characterized for several years as a much smaller biomass than the intestinal one, is constantly changing in the situation of respiratory disorders and is immunomodulated by the intestinal one, on the gut–lung axis.

A material reviewed by Dumas et al. [227] highlights the beneficial role of commensal bacteria in the body in acute viral diseases of the respiratory tract and presents evidence of the contribution of bacteria to local immunity of the lungs or gut.

### 5.3. Probiotics and COVID-19

Recent studies show that although SARS-CoV-2 infection is a disease with initial respiratory manifestations, there are data that revealed the close relationship between the intestinal microbiome and the severity of clinical manifestations in patients with COVID-19.

In a cohort study in two hospitals, per 100 patients with laboratory-confirmed SARS-CoV-2 infection, conducted by Yeoh et al., the compositions of the intestinal microbiome were evaluated by shotgun-sequencing total DNA extracted from stools, as well as the levels of inflammatory cytokines and biological markers. Commensal bacteria with immunomodulatory potential (*Faecalibacterium prausnitzii*, *Eubacterium rectale*, *Bifidobacterium*), were underrepresented and correlated with the severity of the infection, elevated levels of cytokines and inflammatory blood markers (CRP, LDH, aspartate aminotransferase, and gamma-glutamyl transferase). Maintaining the imbalance of the intestinal microbiota (dysbiosis) after the cure of the acute viral infection could be the cause of persistent and long-lasting COVID symptoms [228].

Balancing the intestinal microbiota during and after viral infections can be achieved with the help of probiotics that adhere and line the intestinal mucosa, constituting a strong barrier against pathogens and at the same time, activate the immune system.

It is known that the intestinal microbiota acts on alveolar macrophages and on the intestine-lung axis and develops a defense system against bacterial and viral infections [229].

When an infection occurs in the lungs, the alarm signals are transmitted from the lung to the intestine on the lung-gut axis and from there, the information is transmitted further to the central nervous system (brain) on the gut–brain axis, to stop the inflammatory processes. These data are processed in the cerebral cortex and sent back on the brain–lung–intestine axis, so that the defense processes are implemented; in this way, the microbiota, through its bacterial complexity, mobilizes itself to defend the lung.

Medical research highlights the existence of complex functional connections between lungs and brain, specialized cells transmitting nerve impulses-mediated communication, as an entity made up of related parts via neuroendocrine, immune, and inflammatory networks, the gut–brain–lung axis [230].

Pathophysiology of lungs and intestines is intricately linked, so that an abnormal function in any of them will cause the installation of the disease in the other. The bidirectionality on the lung–intestine axis is accomplished through the products of the microbial metabolism and endotoxins from the gut that reach via bloodstream the lungs, and vice versa, the products of the inflammatory processes in the lungs, will act on the intestinal microbiota [231].

Probiotics act as immunomodulators, stimulate the protection of the host, and can affect the occurrence and severity of disorders at a distance from the intestine.

Oral probiotics have been shown to control respiratory immune reactions.

Probiotics and their mechanisms of action in the prevention and treatment of respiratory diseases, could bring great benefits in the COVID-19 pandemic.

In an experiment conducted by Harata et al. on BALB/c mice infected with influenza virus IFV A/PR/8/34 (H1N1), who were administered intranasally the probiotic LGG, it was found that LGG reduced the respiratory symptoms, increased survival rate compared to the control group and improved the immune responses by increasing the activation of natural killer (NK) lung cells [232].

Severe lung infection with SARS-Cov-2 that binds to ACE2 receptors in lung epithelial cells, has effects also on the intestinal microbiota, by binding of the virus to ACE2 receptors on the enterocytes of the small intestine, so that the SARS-CoV-2 RNA was found in the stool of the infected patients.

Given the bidirectional transmission of information on the gut–lung axis, complex interventions through prebiotics, probiotics, postbiotics, parabiotics, synbiotics, and a personalized diet could modulate the microbiome, improve the immune system activity, and save lives, especially in the elderly and/or debilitated, people with low immunity [231].

De Marcken et al. investigated the activation and response of human blood CD14+ monocytes to single-stranded RNA viruses as being virus-specific and differentially involving the Toll-like receptors (TLRs), TLR7 and TLR8, which triggered different signaling pathways in monocytes, well correlated with the production of cytokines involved in the polarization of CD4+ T-helper cells.

Also, only TLR7 stimulated Ca2+ influx that impede the type-I IFN responses. This study reveals the different signaling pathways activated by TLR7 and TLR8 in human monocytes promoting distinctive T-helper and antiviral replies and specific characteristics during RNA virus infection [233].

After infection with the RNA virus SARS-CoV-2, the body responds through the innate defense system (TLR) that is activated, and through inflammatory pathways, as a defense shield (NLRP3 and NF-κB). Set in motion TLRs trigger the first-incidence antiviral reactions through MYD88—the canonical adapter for inflammatory signaling pathways downstream of members of the Toll-like receptor, and IRF3/7-connected type-I IFN production. As a response to infection and cellular damage, the inflammasome NLRP3, a particular constituent of the innate immune system, coordinates the activation of caspase-1 and the release of pro-inflammatory cytokines IL-1β/IL-18, and under the action of the latter are activated T-cells and macrophages that will secrete IL-6 and TNFα. The IL1B, IL18, IL-6 and TNFα transform supplementary other naïve T-cells into Th1/CTLs/CD8+ or Th17, which generate pro-inflammatory cytokines IFNγ and IL17 [234].

Native and acquired immune responses against infectious viral agents of the respiratory tract are supervised on the bidirectional gut–lung axis by the intestinal microbiome [235].

NF-κB, activated by NLRP3 or TLR4 and the stress-induced mitogen-activated protein kinase (MAPK or MAP kinase) signaling pathway, assists the generation of pro-inflammatory cytokines and apoptosis in enterocytes, but also in lung tissues. Elements resulting from the destruction of tissues following the conflict with the pathogen promote the activation of the innate immune system and an uncontrolled and excessive release of pro-inflammatory signaling molecules, the cytokine storm, i.e., the sudden release in large quantities of cytokines, which can cause multisystem organ failure and death [236].

Some probiotics have been shown to balance the activity of the immune system and inhibit the secretion of pro-inflammatory cytokines, with special implications in the management of COVID-19 and the cytokine storm induced by SARS-CoV-2 infection in severe cases [237].

Kwon et. al. investigated the effects of a cocktail of five probiotics, *L. acidophilus*, *L. casei*, *L. reuteri*, *B. bifidium* and *Streptococcus thermophilus* that proved to be capable of up-regulating the CD4+ Foxp3+ regulatory T-cells (Tregs), to diminish the degree of responsiveness in T-cells and B-cells, and down-regulated T-helper (Th) 1, Th2, and Th17 cytokines, without provoking apoptosis. The probiotics increased the number of dendritic cells with regulatory properties that expressed high levels of IL-10, TGF-β, COX-2 and promoted the generation of regulatory T-cells, also rising the suppressor activity of naturally occurring CD4 + CD25 + Tregs [238].

Recent literature draws attention to the beneficial effects of oral probiotics in preventing and modulating the severity of clinical manifestations of viral respiratory infections.

In the current stage of the COVID-19 pandemic, when there are still no specific drugs for SARS-CoV-2 infection, it would be especially useful to administer known probiotics with antiviral action proven by randomized and placebo-controlled clinical scientific studies.

Studies are needed on the use of probiotics with the concomitant administration of prebiotic oligosaccharides (e.g., fructans, galactans) with the role of enhancing the probiotic strains and balancing the host microbiota [239].

## 6. Photobiomodulation Applied on the Gut–Lung–Brain Axis

Scientific basis for the use of light in clinical medical applications originated at the beginning of the last century in Niels Ryberg Finsen’s first successful experiments [240] on smallpox in red light (1893) and further in 1895, on the treatment of *Lupus vulgaris* (also known as tuberculosis luposa [241]), i.e., painful cutaneous tuberculosis skin lesions with nodular appearance.

Finsen’s ideas and research were promoted and published, and as acknowledgement of his special merits, he received the Nobel Prize in Medicine in 1903 “in recognition of his contribution to the treatment of diseases, especially lupus vulgaris, with concentrated light radiation, whereby he has opened a new avenue for medical science” [242].

Presently, lots of therapeutic techniques that employ low-level laser or LED light limited to a specified set of wavelengths from red to near-infrared, and for some special applications even ultraviolet (UVB), proved to be safe, with no known side effects, used to relieve pain or to heal wounds, ulcers, and to treat many different diseases and disorders under the term of photobiomodulation or PBM, as it stimulates and enhances cell function.

Although high-power lasers are used in surgery and in dermato-cosmetology for cutting or vaporizing tissues, phototherapy using photosensitizers has also been applied in the treatment of tumors as PDT (photodynamic therapy).

In 2008, Santana-Blank et al. highlighted the importance of restoring disturbed physiological rhythms by applying energy through light i.e., photobiomodulation, to bring back the homeostasis–homeokinesis in higher biological systems [243].

About a century later after the scientific work of Finsen, the 2017 Nobel Prize in Physiology or Medicine was awarded to Hall, Rosbash and Young “for their discoveries of molecular mechanisms controlling the circadian rhythm” [244].

Circadian clocks have proven to be particularly important for human physiology adapted to the light-dark cycle of 24 h, so that a person’s sleep habits, eating patterns and diet can desynchronize the body’s clocks and can contribute to the onset of non-communicable diseases [245,246].

External optical signals captured by optical photoreceptors are processed and activate the expression of circadian genes in the central nervous system, influencing molecular clocks and having major implications for some diseases [247].

The spectral quality of the sun modulates our neurotransmitters, and our health suffers because contemporary life often lacks strong daily circadian stimuli [248].

Research has scientifically proved that changes in the monoaminergic neurotransmission in the brain underlie seasonal variations in mood, behavior, and affective disorders [249].

Light could be an allosteric controller for all life-forms, because as Hamblin et al. concluded in a recent review “all life-forms respond to light” [250]; so, light could be able to establish oscillating patterns in our proteins and our organs and could have an impact on our daily rhythms, considering “the seemingly simple, but powerful, idea that repetitive low-energy forces of certain parameters can profoundly affect human physiology” [247].

Abscopal effect (a term derived from the Latin “a scopum” which means “away from the target”), was observed and named in 1953 by Mole, when irradiating the tumor of a mouse on one side of the body, noticed with surprise that a tumor on the opposite side, untreated, shrank [251].

The exact biological mechanisms for the abscopal effect are still being explored, the best hypothesis being the synergistic interaction between the electromagnetic radiation and the molecular and cellular immune network.

As in the general initiation of the immune response against various antigens from bacteria, viruses, fungi, etc., this remote effect can be explained by the initiation and activation of immune cells against antigens [252]. Dendritic cells and macrophages recognize, process, and present these antigenic products on their surface, to be recognized by cytotoxic T lymphocytes, to which they will provide the relevant information for activation, recognition, and killing.

These cytotoxic T-cells trained in this way will circulate at a distance through body fluids, thus having all the information necessary to destroy the other harmful cells of that type, in other body areas which have not been exposed directly to electromagnetic radiation.

Accordingly, the increase of these specific cytotoxic T-cells has been demonstrated to be well correlated with the abscopal responses in irradiated patients, an effect that vanishes after depletion of these cells [253,254,255].

These abscopal responses secondary to the irradiation process are most often obstructed by the immunosuppressive components in the irradiated area, which prevent the proper training of the cytotoxic T-cells, so that they are seldom observed in the clinical practice in patients undergoing solely radiotherapy.

Contrariwise, the association between immunomodulatory medication and the local radiotherapy, may to a certain extent reset the systemic inhibitory immune reactions against the tumor growth [256].

Based on these observations, we can say that PBM, through the abscopal effect could increase the immunomodulatory potential of probiotics or products designed for this purpose.

Just as no rules has been established to date regarding the recommended dose of probiotics in various pathologies, an optimal combination of PBM and probiotics could have a significant effect through the abscopal effect, for which randomized multicenter placebo-controlled studies are still required.

It has recently been shown that the human microbiota contains only 1.3 times more bacteria than the cells of our body (“with an uncertainty of 25% and a variation of 53% over the population of standard 70 kg males”) [257], but still in an impressive number! Some authors consider that: “Humans are superorganisms whose metabolism is a fusion of microbial and human attributes” [258].

The famous physician of ancient Greece, Hippocrates (b. 460 BC, Kos, Greece–d. 370 BC, Larissa, Greece) stated: “All disease begins in the gut” [259]. Today through the advances of evolutionary molecular genetics for the analysis of the intestinal microbiome, we can better understand how 99% of our genes, which are microbial through co-evolution, can affect our immune system and state of health. New promises for the treatment of the chronic diseases opens through recent studies that claim that through diet and a healthy way of living we can modify not only the expression of the human genome, but also the intestinal microbiota [260].

The interplay between the microbiome and the central nervous system has given birth to a new attractive field of scientific research—that of neuromicrobiology. This domain studies the activity of the intestinal–brain axis and the correlation between the microbiome and related pathologies [261], as well as looking to design active applications to fit the effects of the microbiome on the central nervous system, such as for example the probiotics, photobiomodulation, and the brain, in humans or animal models [262].

The whole picture of the connections between microbiome influence and human health is underway to be elucidated.

There is a permanent feedback between probiotics, microbiota, immune system, neuroendocrine, and nerve cells, the intestine–brain axis being significant.

Reasonable dietary fiber consumption and probiotics improve the balance of the intestinal microbiota by stimulating the production of short-chain fatty acids, important for the body’s energy and inflammatory reactions and response, regulating hunger, nutritional status, and body weight, insulin response and energy storage in the liver and muscles [57].

The pioneering of laser medicine (low-level laser applications that is, current photobiomodulation or PBM), is due to the doctor Endre Mester (1903–1984) who immediately after the discovery of the first operational laser, began in 1967 his applications on cutaneous neoplasms; and thus, he discovered the positive biological effects, which were then used successfully in the alternative treatment of various medical conditions [263].

PBM uses especially red to near-infrared radiation to initiate a cascade of events, whose possible mechanisms of action proposed so far are:

- absorption of photons by the first absorbing chromophores, cytochrome c oxidase in mitochondria and non-mitochondrial receptors, such as the ion channels and NADPH oxidase in cell membranes, also with a direct influence on the cellular cytoskeleton [264].
- increased production of ATP, nitric oxide, a sudden outbreak of reactive oxygen species and the modulation of calcium levels.
- initiation of intense generation of transcription factors, synthesis of new proteins, enhanced cell survival, multiplication, and migration.

Depending on the dose of radiation (light) applied, the cellular response will be different, so that low doses of energy will have beneficial, positive, stimulating effects; and, exactly the opposite, for high doses, which will be inhibitory; so, this is the biphasic dose response.

PBM can modulate oxidative stress (in certain cells with low ROS levels, it increases ROS synthesis; and in other cases, it reduces the oxidative stress), reduces the reactive nitrogen species, the prostaglandins levels, and it regulates the NF-kB pathway, it decreases the inflammatory markers in activated inflammatory cells, leading to an overall reduction in inflammation, effects particularly important for respiratory dysfunctions, musculoskeletal system disorders, brain and intestinal tract [265].

Bicknell et al. recently studied the influence of photobiomodulation as local treatment (PBMT) applied directly on the abdomen, to discover possible changes in the composition of the microbiome in mice [266]. This study started from the hypothesis that the microbiome can be modified by food, probiotics, and fecal transplants, which bring microstructures with beneficial effects on health. The group of mice underwent low-power laser therapy in the red (660 nm) or infrared (808 nm) range applied directly to the abdomen, either as single or multiple doses, for a period of 2 weeks.

Fecal aseptic was taken from the intestine before each laser treatment (day 0) on day 7 and day 14; feces were stored at −50 °C until DNA extraction was performed.

The genomic DNA extracted from the fecal pellets was made by the pyro-sequencing technique for the 16S rRNA gene. In this study, the authors demonstrated a significant difference (*p* < 0.05) in microbial diversity in PBM-treated mice compared to control group mice.

This study, even if performed in a small batch, showed for the first time that PBM can influence the diversity of the intestinal microbiota, and can increase the percentage of *Allobaculum*, a bacterium in the category of the good ones in the intestine.

If this treatment with PBM applied also to humans works, then it will open a wide perspective for complementary therapies in various pathologies, such as obesity, neurodegenerative, cardiovascular diseases, and infections such as Coronavirus infection (Covid-19) [266].

Modifications of the intestinal microbiome under PBM therapy, or by other means such as probiotics, will have a targeted effect on the host and the intestinal–brain axis, and will affect the well-being and health status, the stress, and the disease condition, because on this axis circulates information from the enteric, sympathetic, and parasympathetic nervous system to the cerebral cortex and vice versa [267].

One of the disturbing pictures for the modern era is neurodegenerative diseases, which appear after massive neuronal death, i.e., Alzheimer’s and Parkinson’s diseases, increasingly frequently in recent years, for which there are still no satisfactory treatments.

The latest therapies address the signs and symptoms, and only slow down neurodegeneration, but cannot stop it.

PBM has been shown to be an effective and successful alternative—a disease-modifying treatment that stops neuronal destruction, especially when applied in the near-infrared range, for treatment of deficiency in the amount of oxygen reaching the brain, harmful products, genetic alterations, and mitochondrial impairments in degenerated neurons [268].

In the pathogenesis of PD, there are numerous clinical and pathophysiological studies that motivate the hypothesis that the onset of this disease has its origins in the dysfunction of the gut microbiota; information is transmitted via the gut–brain axis through trans-synaptic connections from cell to cell, on the ascending pathways of the sympathetic and parasympathetic nervous system to the substantia nigra and the central nervous system [269].

PBM could correct the disturbances in the mitochondrial energetic metabolism of the intestinal neurons, adjusting the synaptic transmission and the cell secretion, and reinstalling the correct bidirectional transfer of information on the gut–brain axis; so, PBM is a recent and very promising non-pharmacological treatment modality for dysfunctions and diseases on this axis [262,270].

It is known that any history of intestinal infection will increase the resistance of the microbiota to subsequent infections. Thus, the microbiota secures the host from infectious invasions through the so-called colonization resistance; however, the exact mode in which this cardinal evolutionary development occurs is not yet well understood. In time, this operational change is connected with the adjustment of the bile acid metabolism to an increased taurine production. The infectious stimuli potentiate the host taurine production and the extension of taurine consumers. The gut microbiota converts taurine to sulfide, conducting to the amplification of taxonomic group that use the sulfonic acid taurine, and so inhibiting the pathogens respiration. Synthesis of taurine that occurs after the first infection will become a valuable nutrient to feed and train the microbiota in defense against subsequent infections. It has been shown that even the administration of taurine from outside the body is sufficient to induce this change in microbiota function, providing the long-term resistance to infections [271].

Very recently, it was demonstrated by a high-speed flow cytometry screening experiment, single-cell RNA sequencing, and CRISPR–Cas9-based cell-specific in vivo genetic disturbances in mice that there is a set of astrocytes in the central nervous system that can limit inflammation by inducing T-cell apoptosis, receiving signals from the gut bacteria that stimulate anti-inflammatory activity under the action of interferon-γ (IFNγ) produced by natural killer meningeal cells, in which IFNγ expression is modulated by the intestinal microbiome [272].

The commensal microbiota adjusts the host’s defense against pathogenic micro-organisms and the emergence of infectious diseases, and through “co-immunity”, the body is protected not only by its own immune system, but also by the constituents of its microbiota.

The relationship between the intestinal microbiota, the ability of the human body to maintain internal equilibrium by adjusting its physiological processes and the initiation of the diseases at a distance is an assiduous concern of researchers around the world.

If the links between the intestinal microbiota on the intestinal–brain axis are intensively studied, the connections on the gut–lung axis (GLA) are less investigated [273].

Since the discovery of the pulmonary microbiota, it has been shown that there is a strong relationship between the pathophysiological mechanisms and the occurrence of many acute or chronic respiratory infectious diseases. In the lungs, the interactions of micro-organisms (bacteria, fungi, phages, viruses, etc.) are multiple and influence in both directions the immune response; deciphering the pathophysiology of lung diseases and the connection with the microbiota is a promising tool for improving therapeutic protocols [274], in which probiotics play an important role.

From birth and throughout life, there is a strong proved relationship between the structure of the microbiota in the intestine and in the lung [275].

Scientific studies conducted by Madan et al. [276], as well as by Liu et al. [277] demonstrated that changes in infant diet influenced the structure of the microbiota in the lung, and experimental research on fecal transplantation in rats pointed out changes in the lung microbiota.

Trompette et al. demonstrated the effects of fermented dietary fibers with anti-inflammatory properties due to SCFAs on influenza-infected mice, which increased their survival through two interrelated mechanisms. Mice fed a high-fiber diet (HFD) had an improvement in the medullary hematopoietic activity, objectified by rising the number of monocytes, which led to an increase in the number of activated macrophages, reducing the production of CXCL1 chemokines [chemokine (C-X-C motif) ligand 1] in the respiratory tract, with an important role in regulating the immune and inflammatory responses. Decreased CXCL1 secretion lowered the recruitment of airway neutrophils, thus reducing immunopathological processes during influenza infection. At the same time, SCFAs had activated the CD8+ T lymphocytes. Dietary fermentable fibers and SCFAs remitted influenza infection, favorably improving the balance of innate and adaptive immunity [278].

Changes in the intestinal microbiota induced by antibiotic therapy in the neonatal period may underlie the onset of asthma in childhood, because it is associated with a disturbance of fecal SCFAs concentration [279].

In the human body there are direct and indirect relationships between different parts of the microbiota, which adapt to each other at the level of each organ. The imprint of the intestinal microbiota acts on immune system both locally (in the gut), and at long distance (in the lungs) by involving many cells, cytokines (CD8+ T-cells, Th17, IL-25, IL-13, prostaglandin E2), and/or NF-κB-dependent signaling pathways.

Pulmonary microbiota also influences the mucosal immunity, participating in immune tolerance by activating neutrophils and secreting pro-inflammatory cytokines, facilitated by receptor 2 (TLR2), with the release of β-defensin 2 antimicrobial peptides activated by helper T-cells (Th17).

At the same time, immune signals are transmitted from the lung microbiota to the communities of commensal, symbiotic, and pathogenic micro-organisms in the gut, through insufficiently elucidated immune mechanisms, associated with the presence of the Th17 lymphocytes in the case of lung influenza viral infection. Both microbiota (intestinal and pulmonary) can be modified by diet, medication, and probiotics. At the level of the intestine—lung axis, there is a permanent and bidirectional involvement and interaction between the microbiota and the immune system, which influences the health or disease of the host [280].

## 7. Photobiomodulation and COVID-19

RNA viruses include a multitude of viral agents that put a great pressure and significant public health alarms worldwide, especially when generate human pandemics and lethal threats.

The following well known RNA viruses that cause human maladies are influenza virus, rhinovirus, respiratory syncytial virus, rotavirus, measles virus, hepatitis C virus, human immunodeficiency virus, Ebola virus, Zika virus, dengue virus, yellow fever virus, poliovirus, SARS coronavirus etc.

The scientific research for mastering all the pathophysiological mechanisms in viral infections will lead to the judicious projects for effective and safe vaccines, whereas the therapeutic strategies against these viruses has become an increasing priority in the medical field for all the nations around the globe [281].

In the new era of coronaviruses that began in recent decades, a new frightening pandemic broke out in December 2019 in Wuhan, China, triggered by the new coronavirus called SARS-CoV-2 (Severe Acute Respiratory Syndrome Coronavirus 2) that induced the viral infection named Coronavirus Disease 2019 (COVID-19), which affected the whole globe and for which there is still no satisfactory treatment, more than 2.475 million people worldwide died from it [282].

After contacting the viral infection, the clinical manifestations begin on average in 5 days with mild, moderate, or severe symptoms, which include: rhinorrhea, nasal itching, anosmia, loss of taste, headache, fever, diarrhea, malaise, myalgias, insomnia, cough torturous, tiring, ineffective, dyspnea with polypnea, generalized cyanosis, respiratory failure, heart failure. In some severe cases, the patient may eventually die.

During an intense inflammatory process, such as SARS-CoV-2 infection, severe vasodilation occurs and intestinal permeability increases, so that bacteria in the gut can easily cross this barrier (“leaky gut”) to the lungs, where they will encounter the same congestion with alveolo-capillary vasodilation. In this way, the lungs become loaded with intestinal bacteria (Bacteroidetes and Enterobacteriaceae, a phenomenon called “more intestine in the lungs”), which will cause a hypersecretion of cytokines, accelerating even more strongly the inflammatory process, infection and acute lung damage and causing acute respiratory distress syndrome (ARDS). The intensity of activation, but especially the concentration of angiotensin 2 conversion enzyme (ACE2) in the lungs and intestines (because ACE2 is located more on the luminal surface of intestinal epithelial cells), will influence the clinical picture, the response to therapy, and the subsequent evolution [283].

SARS-CoV-2 has a special affinity to ACE2-receptors of the epithelial cells in the breathing airways, producing systemic hyperinflammation in severe cases.

The inflammatory process is systemic and cause vasodilation with lymphocyte and monocyte infiltrate into the lungs and heart. Activated T-cells secrete colony-stimulating granulocyte macrophages (GM-CSF) and in turn by chemotaxis recruit monocytes with strong pro-inflammatory potential through excessive secretion of IL-6, phenomena that will correlate with severe lung disease in some patients with COVID-19 [284].

Paraclinical data show leukopenia, lymphopenia, thrombocytopenia, hypoalbuminemia, alarming increase in C-reactive protein (CRP), serum ferritin, aminotransferase, lactate dehydrogenase (LDH), D-dimer and decreased alkaline reserve, and so on, implying a particularly severe immunopathology.

Study of immunological parameters reveals increased concentrations of pro-inflammatory cytokines interleukin 1 beta (IL-1β), IL-2, IL-6, interferon-gamma (IFNγ), interferon-gamma-inducible protein-10 (IP10) and monocyte increase in serum chemotactic protein-1 (MCP1), with a reduction in CD8+ T lymphocytes (often called cytotoxic T lymphocytes, or CTLs) and an increase in the number and function of T-helper type 1 (Th1) lymphocytes.

At the same time, the activation of type 2 helper T lymphocytes (Th2) takes place and increase the secretion of the followings: IL-4, IL-7, IL-8, IL-9, IL-10, fibroblast growth factor (FGF), granulocyte colony-stimulating factor (G-CSF), GM-CSF, macrophage inflammatory protein 1A (MIP-1A) and 1B (MIP-1B), platelet-derived growth factor (PDGF), TNF-α, and together with elevated serum levels of VEGF (vascular endothelial growth factor). This exaggerated synthesis of pro-inflammatory factors, increased levels of the C-reactive protein, fibrinogen, and platelets were considered an immunological storm in COVID-19. Elevated levels of interleukin-6 (IL-6), recognized as the major mediator of the inflammatory and immune response initiated by viral infection, have been observed in over 50% of patients with COVID-19 and have been associated with respiratory failure, the need for mechanical ventilation and/or intubation, and high mortality in these severe forms [285].

Photobiomodulation through its remote molecular and cellular effects could modulate the mechanisms of the cytokine storm by reducing local and systemic inflammatory responses on the gut–lung–brain axis.

**Figure 1:**
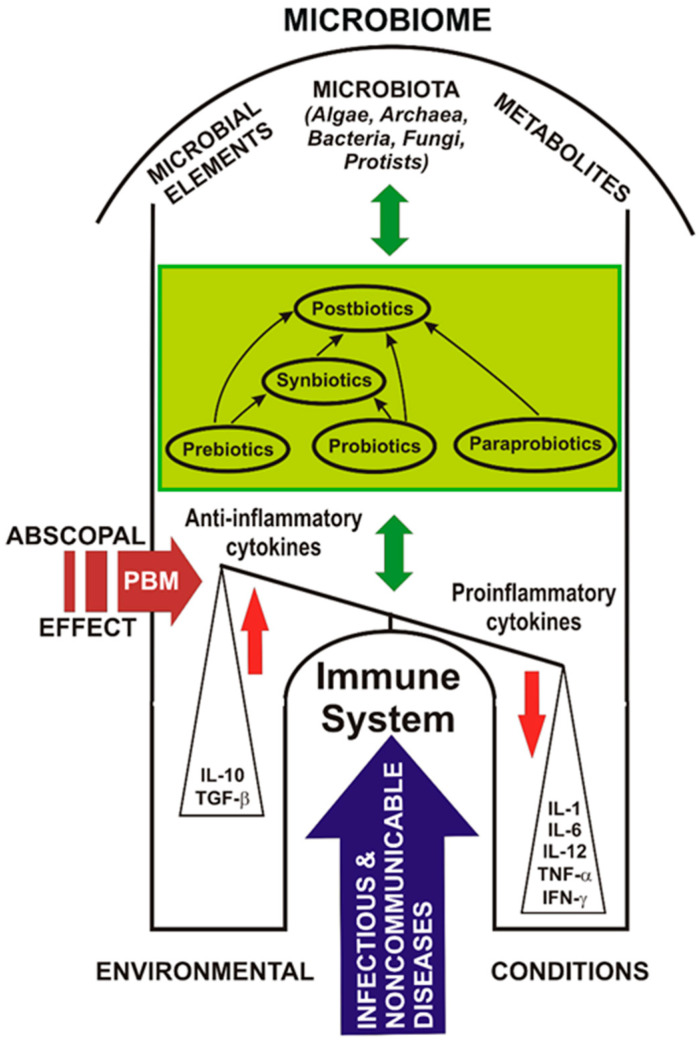
Probiotics, photobiomodulation, and the microbiome in COVID-19.

Coupled complex PBM and probiotic interventions can adjust the microbiome, improve the activity of the immune system, and save the lives of people with immune imbalances, as in the model suggested in Figure 1.

Figure 1 shows a model of the abscopal effect of PBM on the human microbiome and the relationship between probiotics, the immune system, and diseases affecting the host.

Probiotics may have the ability to modulate exacerbated immune responses, such as the COVID-19 cytokine storm. Targeting the SARS-CoV-2 cytokine storm using PBM and probiotics could be a useful treatment choice.

In the case of COVID-19, PBM could influence the balance between anti-inflammatory and pro-inflammatory cytokines, leading to the resolution of the infectious disease.

In a study conducted by Mehani in 2017, there were compared the immunomodulatory effects of inspiratory muscle training (IMT) and photobiomodulation [level laser (LLL) acupuncture stimulation for about 8 weeks] in patients with chronic obstructive pulmonary disease on interleukin-6 (IL-6) and the lymphocytes CD4+ T/CD8+ T. The results proved the reduction in plasma IL-6 concentration, and the increase in CD4+/CD8+ ratio, with a superior effect of photobiomodulation over IMT in the adjustment of the immune lung inflammation. PBM is efficient in rising CD4+ and CD8+ T-cells and makes improvements to their equilibrium [286].

Diao et al. analyzed the total number of T-cells, CD4+ and CD8+ T-cells, which were dramatically reduced in COVID-19 patients and negatively correlated with their survival. Total number of T-cells were also negatively correlated with serum IL-6, IL-10, and TNF-α concentration, and in the case of favorable evolution were found decreased IL-6, IL-10, and TNF-α concentrations and total number of T-cells was reestablished. Research has shown that for patients with a total T-cell count below 800/μL, even without severe symptoms, immediate intervention is required, as their condition could worsen very quickly [287].

Cury et al. treated the acute lung injury in C57BL/6 mice with LLLT (660 nm, radiant exposure of 10 J/cm^2^) and obtained the decrease of expression and secretion of cytokines (TNF-α, IL-1β, IL-6,) and chemokine (MCP-1). This study proved that PBM could play an essential role in controlling the immune reactions (the polymorphonuclears, monocytes, macrophages, the pro-inflammatory cytokines, and collagen deposition), inducing an important drop in both inflammatory cell influx and inflammatory mediators’ secretion, so that PBM is efficient in decreasing the inflammatory reactions in lungs and in promoting the pulmonary tissue regeneration [288].

Increased levels of IL-1β (central role in the initiation of the inflammatory processes), IL-6 (pleiotropic cytokine, increased in the lungs and plasma) with key role in acute respiratory distress syndrome (ARDS) pathophysiology, and IL-8, proved to be linked with persistent inflammation and poor prognosis in ARDS patients. PBM significantly reduced the severity of ARDS by decreasing the IL-1β, IL-6 (both in the lungs and plasma), and IL-8 (in the lungs), also lowering the mortality rate [289].

It is well accepted that PBM is a noninvasive treatment method which reduces inflammation and stimulates tissue regeneration and healing processes [290].

PBM could be used in the control of pathophysiological mechanisms in SARS-CoV-2 viral infection, especially in the acute respiratory distress syndrome, the modulation at long distance of the immune system and increased oxygenation of blood flow, but also in the subsequent symptoms post-COVID, which are annoying and last for months in some patients discharged.

Any feasible therapy should be applied in the current severe pandemic, if it can reduce the inflammatory processes triggered by viral infection in the lungs, reduce edema and bronchoconstriction, stop the degradation of the alveolo-broncho-pulmonary structures, permeabilize the airways to ensure the oxygen supply, and finally to restore the normal respiratory function.

In an experiment conducted by Maldaner et al. on H_2_O_2_-induced inflammation of skin fibroblast cell line (HFF-1), photobiomodulation (660 nm, 3–8 J/cm^2^) partially reversed the activation of DNA oxidation, caspase 3/8, IL-1β/6 and IFN-γ induced by H_2_O_2_, with an increased level of anti-inflammatory IL-10 at an energy density applied of 4 J/cm^2^ (*p* <  0.001), which also improved the cellular proliferation for the fibroblasts treated with H_2_O_2_ and exposed to LLLT [291].

Human infectious diseases have a fluctuating profile of symptoms and resolution, initially through acute manifestations that may regress, or worsen through a mixed profile included in the pro-inflammatory immediate small-scale environment cytokines (governed by IL-1β, IL-6, IL-12, IL-23, and TNF-α) and tissue injuries caused by type M1 activated macrophages, and wound healing operated by type M2 alternately activated macrophages in an anti-inflammatory environment (dominated by IL-10, transforming growth factor (TGF)-β, chemokine ligand (CCL) 1, CCL2, CCL17, CCL18 and CCL22), evolving back and forth between the analogous extremes M1 and M2, due to the intricate puzzle during infectious diseases [292].

Macrophage is an important mediator of inflammation: M1 phenotype is the pro-inflammatory type for direct host-defense against pathogens, while M2 phenotype is involved in the resolution phase of inflammation and tissue repair [293].

In an experimental study, Carvalho et al. investigated the effect of phototherapy (PhT) on lipopolysaccharide-activated (LPS) cells from *E. coli*, known to control the release of inflammatory mediators from various LPS-activated cells. They used U937 cells, a line of human monocytic cells, cultured and matured to macrophages in an LPS medium and irradiated (660 nm) at 4.5 J/cm^2^. The experiment proved that pro-inflammatory cytokines and chemokines, ROS and NF-κB were down-regulated by PhT, while IL-10, arginase, PGC-1β and glutathione were up-regulated. The Sp1 activity increased after PhT to values higher than those from cells only LPS-treated. Finally, PhT restored the polarization of macrophages to the M2 model, as well as balanced oxidative stress and modulated the immune response by regulating IL-10 secretion through a mechanism in which the transcription factor Sp1 plays a crucial role [294].

Therefore, M1/M2 ratio and the oxidative stress could be adjusted by photobiomodulation in human macrophages, with important clinical consequences in the disease’s management. PBM is a technique capable of influencing polarization to determine the transformation between activated macrophages M1 (pro-inflammatory) to turn into M2 (anti-inflammatory) and could be an extremely valuable adjunctive method for resolving inflammation in the lungs affected by SARS-CoV-2.

Type-I interferons (IFN-I) include a set of signaling proteins known for their strong antiviral action.

IFN-I are secreted by most cells and comprise IFN-α, IFN-β, IFN-ο, IFN-δ, IFN-κ, IFN-ε, IFN-τ, and IFN-ω, which directly interfere a powerful antiviral response [295].

Release of IFN-I take place in essence when the pattern recognition receptors existing on the cell membrane or in the intern cytosolic department of all cells, are activated by the pathogen-associated molecular patterns, of which the most investigated are Toll-type receptors [296].

IFN-γ is a cytokine produced by the natural killer (NK), CD4+ T and CD8+ T-cells, and has a particularly important role for its antiviral effects by inducing IFN genes, as well as by modulating the immune response to infection [297].

Immunomodulatory effects induced by IFN-γ are achieved by activating and differentiating immune cells, as well as by the direct intracellular antiviral effect [298].

An experimental in vitro research on isolated immune cells from patients with multiple sclerosis (MS) and healthy donors, studied the effects of PBM therapy with three wavelengths (670 nm, 735 nm and 830 nm) on cytokine production by the immune cells. PBMT with 670 nm reduced the clinical severity of MS by decreasing of oxidative stress, pro-inflammatory cytokines, and the death of cells; it also increased the production of anti-inflammatory cytokines. Both wavelengths (670 nm and 830 nm) increased IL-10 and reduced IFN-Ɣ in cells from MS, finally demonstrating a differential regulation of the immune response in MS patients and healthy donors [299].

PBM stabilize the function of the immune system (drop the level of pro-inflammatory cytokines such as IL-1β, IL-6, IL-8, TNFα, and MCP-1 and improve the balance of IL-10) in severe COVID-19 cases, decreasing the impact of cytokine storm as the main cause of high mortality in ARDS patients [300].

Extracellular matrix (ECM)-derived platforms supplied the excitement to expand new regenerative therapies to advance the clinical applications for creative functional grafts in persistent organ failure.

Mesenchymal stem cells could be activated in the presence of interferon-gamma (IFN-γ), IL-8, and IL-1β, as a microenvironment. PBM, as immunotherapy, could control the levels of cytokines and chemokines, and has sterilization effect. Recently, Guimarães et al. have assessed the effect of PBM and continuous positive airway pressure (CPAP) on pulmonary recellularization in the case of decellularized lungs from C57BL/6 mice. Mesenchymal stem cells derived from human tooth pulp and pulmonary epithelial cells (BEAS 2B and A549) were seeded into lungs and incubated PBM at wavelength of 808 nm, 100 mW, for 30s was applied. Culture media were analyzed. The conclusion was that PBM improved all parameters and the recellularization pulmonary process [301].

Summarizing the research, we can highlight that the administration of probiotics is demonstrated in numerous animal studies and clinical models as having beneficial effects on host immunity and exerts protection against the aggression of viral pathogens with encouraging results for the prophylaxis and therapy of respiratory diseases (Table 4).

Table 5 shows the effects of PBM in the action on probiotics [307], followed by a series of experimental animal studies [266,288,289,308,309], in vitro and/or in vivo cellular models [291,310,311,312], including the latest clinical trials in patients with COVID-19 [313,314], which demonstrates the perspective applications of PBM for the targeting and modulation of the microbiome [266,270,308,309], with also the enrichment of the functional genes [308], the immune system [268,270,288], the auxiliary control of chronic degenerative diseases and viral infections [310,312,313,314], as a challenge for future research in the 21st century (see also Figure 1).

## 8. Conclusions

Probiotics, together with a personalized diet, could balance the microbiome and improve the immune system activity of the host.

Probiotics may have the ability to modulate exacerbated immune responses, such as the COVID-19 cytokine storm.

PBM, through its remote molecular and cellular effects, could adjust the mechanisms of the cytokine storm in COVID-19 by reducing local and systemic inflammatory responses on the gut–lung–brain axis.

Coupled complex PBM and probiotic interventions can control the microbiome, improve the activity of the immune system, and save the lives of people with immune imbalances.

According to scientific studies, PBM has an important immunomodulatory and anti-inflammatory role, without side effects, unlike anti-inflammatory drugs such as corticosteroids, which, in addition to the beneficial and even salutary effect in saving lives, cause delayed response to virus elimination, secondary infections, and increase hospitalization in acute viral infections.

The use of well-documented probiotics for viral respiratory infections, together with PBM in the control of the immune system, could reduce the medical, financial, social, and psychological difficulties, and the severity of the loss of so many lives, which is out of control in this pandemic.

In the near future, photobiomics and metabolomics should be applied innovatively in the SARS-CoV-2 crisis (to study and design new therapies for COVID-19 immediately), to discover how bacteria can help us through proper energy biostimulation to fight against this pandemic, managing and succeeding to find the key to the hidden code of communication between RNA viruses, bacteria, and our body.

## Figures and Tables

**Table 1:** Definitions, sources and types of pre-, pro-, para-, post-, and synbiotics [70,71,72,73,74,75,76,77,78,79,80,81,82,83,84,85,86,87,88,89,90,91,92,93,94,95,96,97,98,99,100,101,102,103,104,105,106,107,108,109,110,111,112,113,114,115,116,117].

	Prebiotics	Probiotics	Paraprobiotics	Postbiotics	Synbiotics
**Definition**	Prebiotics are a group of nutrients that are degraded by gut microbiota “dietary prebiotics” as “a selectively fermented ingredient that results in specific changes in the composition and/or activity of gastrointestinal microbiota, thus conferring benefit upon host health” [71].	Live micro-organisms that, when administered in adequate amounts, confer a health benefit on the host, probiotic fermented food [72]. Food fermented by or containing probiotic(s) with strain-specific/without strain-specific evidence [73].	They are also called phantom or inactivated probiotics and are in fact “non-viable microbial cells (either intact or broken), or crude cell extracts that, when administered (either orally or locally) in appropriate amounts, confer a benefit to the human consumer or animal” [70]. (or metabolic byproducts) secreted by viable bacteria or released after their lysis” [74]. As “inactivated probiotics” and “ghost probiotics” [75,104,105,106,107,108,109,110,111,112,113].	They are “non-viable bacterial products or metabolic products” from micro-organisms that have biological activity in the host. [75,104,105,106,107,108,109,110,111,112,113].	Probiotics and prebiotics that are used in combination, are known as “synbiotics” [76].
**Sources**	Asparagus, sugar beet, garlic, chicory, onion, Jerusalem artichoke, wheat, honey, banana, barley, tomato, rye, soybean, human’s and cow’s milk, peas, beans, etc., and recently, seaweeds and microalgae [77].	Fermented natural or industrial products with one or more types of bacteria such as: *Lactobacillus acidophilus*, LGG, *Lactobacillus casei Shirota*, *Lactobacillus gasseri*, and *Bifidobacterium bifidum* Yogurt; Frozen yogurt; Kefir; Buttermilk; Acidophilus milk; Lebne; Viili; Lassi; Aged cheeses; fermented cabbage; Pickles and olives produced by traditional methods [78].	Different species of bacterial cultures: *Lactobacillus* spp. *Bifidobacterium* spp. proved their efficacy after inactivation, especially with heat. Bioactive compounds: *Bifidobacterium lactis* Bb12: peptides and proteins Lactic acid bacteria: peptidoglycans, lipopolysaccharides, and DNA *Saccharomyces cerevisiae*: β-glucan *Lactobacillus* strains: lipoteichoic acids LGG: lipoteichoic acid and peptidoglycans *Lactococcus lactis H61* teichoic acid and lipoteichoic acid [79].	Metabolic byproducts of live probiotic bacteria such as cell-free supernatant, vitamins, organic acids, short-chain fatty acids, secreted proteins/peptides, bacteriocins, neurotransmitters, secreted biosurfactants, amino acids, flavonoids derived postbiotics (desaminotyrosine, equol daidzein, daidzein, norathyriol), terpenoids derived postbiotics (genipin, paeoniflorin, paeoni lactone glycosides, paeonimetabolin I, II, III), phenolic-derived postbiotics (equol, urolithins, valerolactones, enterolactone, enterodiol, 8-prenylnaringenin) etc. [80].	*Lactobacillus* spp., *Bifidobacterium* spp., *S. boulardii*, *B. coagulans* are probiotic strains that are used in synbiotic formulations; whereas the prebiotics used are as: oligosaccharides (fructooligosaccharide (FOS), galacto-oligosaccharide GOS and xyloseoligosaccharide (XOS), and inulin (from natural sources such as chicory and yacon roots)).
**Types**	Fructans Galacto-Oligosaccharides Starch and Glucose-Derived Oligosaccharides Other Oligosaccharides Non-Carbohydrate Oligosaccharides (e.g., cocoa-derived flavanols) [81]. Fermented grain foods or vegetables as well as beer and wine that contain β-glucans, oligosaccharides and polyphenols compounds. [82,83,85].	Fermented natural or industrial products. The most popular types of probiotics are: *Lactobacillus*, or *Döderlein’s bacillus*; *L. casei*; *Bifidobacterium bifidus*; *Saccharomyces boulardii* [86].	Paraprobiotics consist of a wide range of molecules including peptidoglycans, surface proteins, cell wall polysaccharides [87].	Non-viable probiotics Biosurfactants Exopolysaccharides Cell surface proteins Teichoic acids Peptidoglycans Cell-free supernatant and soluble factors Bacteriocins Short-chain fatty acids Vitamins	Combinations of probiotic and prebiotic types.

**Table 2:** Mechanisms of action, clinical applications and side effects of pre-, pro-, para-, post-, and synbiotics [70,71,72,73,74,75,76,77,78,79,80,81,82,83,84,85,86,87,88,89,90,91,92,93,94,95,96,97,98,99,100,101,102,103,104,105,106,107,108,109,110,111,112,113,114,115,116,117].

	Prebiotics	Probiotics	Paraprobiotics	Postbiotics	Synbiotics
**Mechanisms of Action**	Could change the composition and population of the intestinal microbiota [30,31,32,33,34,35,36,37,38,39,40,88]. Anti-inflammatory effects by increasing short-chain fatty acids (SCFAs) [acetate, propionate, and butyrate] [89]. Influences glucose and lipid metabolism [90]. Important role in cell proliferation, differentiation, and apoptosis [91]. Improve immunity functions by increasing the population of protective micro-organisms by: Oligofructose and inulin mixture Fructo-oligosaccharides (FOS), Galacto-oligosaccharides (GOS) Trans-galacto-oligosaccharides (TOS) Can affect the brain by the vagus nerve. It affects the brain through three routes, including neural, endocrine, and immune pathways [71].	a. Colonization and regulation of dysbiotic intestinal microbiota [92]. b. Protection of the epithelial barrier by maintaining tight junction integrity [74,93]. c. Induction of mucin production and B-cell-secreting IgA [94]. d. Ability to increase adhesion to the intestinal mucosa and to inhibit adhesion of the pathogens through competition [95]. e. Administration of antimicrobial products such as acetic and lactic acids and bacteriocins, which have strong inhibitory effects against Gram-negative bacteria [96]. f. Produce of SCFAs with anti-inflammatory and immune modeling effects. Participates in the differentiation, proliferation cell and release of immune pathway signaling molecules. SCFAs increase the expression of the anti-inflammatory cytokine IL-10 and suppress pro-inflammatory responses [65,67,97]. h. Gut–brain axis interaction with the production of metabolites as well g-aminobutyric acid (GABA) [98]. i. Adjusting the innate and / or adaptive immune response of the host [99].	Colonization and regulation of dysbiotic intestinal microbiota. Protection of the epithelial barrier. Ability to increase adhesion to the intestinal mucosa. Produce of SCFAs with anti-inflammatory and immune modeling effects. Immunomodulatory, adjusting the innate and / or adaptive immune response. Inhibition of the NF-kB signaling pathway. Antiviral Antihypertensive Hypocholesterolemic Antiproliferative Antioxidant Immunomodulatory [100]. Anti-inflammatory [101]. Antimicrobial and maintaining of gut health [102]. Antitumor activity Antimicrobial properties Antagonistic effects against pathogens	Immunomodulator, influenced by retinoic acid-acting mucosal dendritic cells and their subsequent effects on regulatory T-cells, with increased IL10 production [103]. Anti-inflammatory action: increases IL-10 secretion, inhibits TNF-α secretion, reduces IL-12, increases IL-8 levels, blocks NF-κB activation. Antioxidant activity Antitumor effects [104]. Infection prevention [105]. Anti-atherosclerotic [106]. Autophagy [107]. Accelerated wound healing [108].	Elevated levels of lactobacilli and bifidobacteria, balance the intestinal microbiota. Prevention of bacterial translocation and the incidence of nosocomial infections in surgical patients. Improving liver function in patients with cirrhosis. Improving immunomodulatory capacity [109].
**Clinical** **Applications**	Irritable Bowel Syndrome Crohn’s Disease Colorectal Cancer Necrotizing Enterocolitis Memory, concentration, and learning; Mood; Autism Allergic skin diseases; atopic dermatitis. Cardiovascular diseases Calcium absorption [71,95].	Prevention and amelioration of various diseases: Acute nosocomial diarrhea, secondary to antibiotic therapy Allergic manifestations (eczema, allergic rhinitis, conjunctivitis, wheezing) Diarrhea due to inflammatory bowel disease Type 2 diabetes Obesity Heart disease Cancer etc. [87,95].	Anti-infective Anti-allergic Obesity Anti-cancer Anti-inflammatory bowel disease Effects on respiratory diseases Recovery of intestinal injuries [70,87].	Treating or preventing multiple diseases: Alzheimer’s disease, Allergies, Inflammatory bowel disease, Multiple sclerosis, In addition, in particular, many diseases in children [70,74].	Are considered important tools in maintaining human and animal health, and in the prevention and/or alternatives to reduce the risks associated with diseases. Improve metabolic dysfunction and prevent diabetes in prediabetes [110]. Obesity [111,112]. Irritable bowel syndrome [113]. Suppression allergy syndrome Prevent asthma [114]. Disease prevention (e.g., prophylaxis of various types of cancer) Manages health. Reducing healthcare costs.
**Side Effects**	Prebiotics have no life-threatening or severe side effects. In some cases, abdominal discomfort, bloating and gas may occur while the digestive system adjusts [71].	“Probiotics” may theoretically be responsible for four types of side effects: 1. Systemic infections 2. Deleterious metabolic activities 3. Excessive immune stimulation in susceptible individuals 4. Gene transfer [40,41,72].	Paraprobiotics have long shelf life, safety, and beneficial effects, such as modulation of immunity, modification of biological responses, reduction of cholesterol, anti-inflammatory, and antiproliferative properties. [79].	(1) There is no risk of bacterial translocation from the intestinal lumen into the blood of vulnerable and immune-compromised subjects (2) There is no chance of acquiring and transferring genes that produce antibiotic resistance (3) Easier to extract, standardize, transport and store (4) Loss of viability through cell lysis can produce additional benefits (5) Improved interaction of each molecule released from cells disrupted with the epithelial cells [115].	Prebiotics and probiotics together tested to date have a strong safety record [116], and synbiotics formulated with them might also be presumed safe for the same intended uses [117]. Mild side effects are gas, bloating, digestive problems such as diarrhea or constipation.

**Table 3:** Side effects of probiotics [130,131,132,133,134,135,136,137,138,139,140,141,142,143,144,145,146,147,148,149,150].

Side Effects	Probiotics	Disease	Brief Description of the Study	Reference
**Bacteremia**	LGG	Ulcerative colitis *Lactobacillus* bacteremia	A case of *Lactobacillus* bacteremia has been described in a 17-year-old boy with ulcerative colitis treated with systemic corticosteroids and infliximab, who had a fever of 102 °F, flushing and chills one week after the start of LGG probiotics.	[130]
	LGG	Severe active ulcerative colitis in an adult patient	It was reported on a case of bacteremia caused by LGG in an adult patient affected by severe active ulcerative colitis under treatment with corticosteroids and mesalazine.	[131]
	LGG	89 patients with *Lactobacillus* bacteremia; 82% of cases had severe or fatal comorbidities	Risk factors and outcome were analyzed for 89 patients with *Lactobacillus* bacteremia. Mortality was 26% at one month, and 48% at one year. Serious underlying diseases were a significant predictor of mortality, while in vitro effective antimicrobial treatment was associated with lower mortality.	[132]
	LGG and 7 different species	Collection of cases of *Lactobacillus* bacteremia, National Infectious Disease Register (NIDR), 1995–2000, Finland	90 cases of *Lactobacillus* bacteremia were diagnosed, of which LGG was the most common species. Annual incidence of *Lactobacillus* bacteremia in the Finnish population was, on average, 0.29 cases/100,000 inhabitants/year.	[133]
**Fungemia**	*Saccharomyces boulardii*	*Clostridioides difficile* acute diarrhea *Saccharomyces boulardii* fungemia	A case of *Saccharomyces cerevisiae* fungemia has been reported in a patient with *Clostridioides difficile*-associated diarrhea (CDAD) in oral treatment with *S. boulardii* and vancomycin. The identification of *S. cerevisiae* was confirmed by molecular technique. Fungemia is a rare but serious complication of probiotic treatment. The authors draw clinicians’ attention to the risk of toxic effects when prescribing probiotics, especially for immunocompromised patients.	[134]
	*Saccharomyces boulardii*	*Clostridioides difficile* recurrent diarrhea. Rheumatoid arthritis. Anemia. Malnutrition *Saccharomyces boulardii* fungemia	The authors published the case of 79-year-old female with rheumatoid arthritis, who after a resection of the intestine developed *S. boulardii* fungemia. She had complications: anemia, malnutrition and several nosocomial infections, including recurrent diarrhea associated with *C. difficile*. Diarrhea was treated with Metronidazole, Vancomycin and Sachaflor (probiotic *Saccharomyces cerevisiae*, subtype *S. boulardii*).	[135]
	*Saccharomyces boulardii*	*Clostridioides difficile* colitis. *Saccharomyces boulardii* fungemia	A case of fungemia caused by *Saccharomyces cerevisiae* in an elderly patient treated orally with *S. boulardii* in combination with vancomycin for *Clostridioides difficile* colitis. It is not recommended the administration of this viable yeast, especially in debilitated patients with active colitis.	[136]
	*Saccharomyces boulardii*	Head and neck cancer Aseptic diarrhea Oral mucositis	65-year-old man who developed *Saccharomyces cerevisiae* fungemia after completing a course of chemotherapy and radiation therapy for head and neck cancer. For grade IV oral mucositis and received *Saccharomyces boulardii* (Perenterol) orally as a treatment for aseptic diarrhea, just before the onset of fungemia.	[137]
	*Saccharomyces boulardii*	Myeloid leukemia *Saccharomyces* fungemia	*Saccharomyces* fungemia in an 8-month-old baby with acute myeloid leukemia during treatment with intensive chemotherapy. Patient received prophylaxis treatment with *Saccharomyces boulardii* (Codex) capsules to prevent diarrhea. *Saccharomyces cerevisiae* was isolated from blood culture, although the patient also received antifungal prophylaxis with fluconazole.	[138]
	*Saccharomyces boulardii*	Vascular catheter *Saccharomyces* fungemia	Four cases of *Saccharomyces boulardii* fungemia in patients who had a vascular catheter. To prevent catheter contamination, the authors recommend that packages or capsules of *Saccharomyces boulardii* be opened with gloves outside the patient’s room.	[139]
**Fungemia**	*Saccharomyces boulardii*	Seven cases of fungal infection with *Saccharomyces boulardii* pathology in Intensive Care Unit	Seven cases of fungal infection with *Saccharomyces boulardii* (Sb) occurred in a 12-bed intensive care unit (ICU); 11 severely ill patients, mechanically ventilated, treated with broad-spectrum antibiotics with central venous catheter and previously treated with Sb. Explanation of the phenomenon was discussed: (1) a high-dose intestinal translocation in seriously ill patients; (2) a contamination of the central venous catheter and (3) a massive colonization of patients with this yeast.	[140]
	*Saccharomyces boulardii*	Pathology in Intensive Care Unit *S. cerevisiae* fungemia	3 patients were identified with *S. cerevisiae* fungemia in an intensive care unit (ICU) after receiving a probiotic containing *Saccharomyces boulardii* (Ultralevura) through a nasogastric tube for an average duration of 8.5 days. A literature review identified another 57 cases of *S. cerevisiae* fungemia, of which 60% of patients were in intensive care and 71% received enteral or parenteral nutrition. The use of probiotics was identified in 26 patients and 17 patients died. The administration of *S. cerevisiae* probiotics should be carefully reevaluated, especially in immunosuppressed patients or critically ill patients.	[141]
	*Saccharomyces boulardii*	Pathology in Intensive Care Unit *S. cerevisiae* fungemia	Two cases of fungemia in an intensive care unit after a probiotic treatment containing *S. boulardii*. The authors draw attention to the use of probiotics in patients with critical illnesses.	[142]
	*Saccharomyces boulardii*	Pathology in Intensive Care Unit *Saccharomyces boulardii* probiotic-associated fungemia	A case of fungemia in an immunocompetent patient after administration of probiotics containing *Saccharomyces boulardii*; the fungal infection was proved by genomic and proteomic modeling methods. Study calls into question the safety of this preventive biotherapy.	[143]
	*Saccharomyces boulardii*	*Saccharomyces cerevisiae* fungaemia	Seven patients with *S. cerevisiae* fungus were reported in two hospitals in India between July 2014 and September 2015. Two patients were premature newborns, and five adults were admitted to an intensive care unit and received probiotics containing *S. boulardii*. Five patients responded promptly to echinocandins or voriconazole. The authors recommend avoiding the probiotic containing *S. boulardii* in patients with critical conditions.	[144]
	*Saccharomyces boulardii*	Urosepsis superinfected with *Klebsiella pneumoniae* and *Escherichia coli* and diarrhea. *Saccharomyces cerevisiae* fungemia	An 88-year-old patient was admitted to the intensive care unit with a diagnosis of urosepsis superinfected with *Klebsiella pneumoniae*, *Escherichia coli* and diarrhea. He received empirical treatment with meropenem (2 × 500 mg) and linezolid (1 × 600 mg), through a central venous catheter (CVC); for the relief of diarrhea received *S. boulardii* (Reflor 250 mg capsules). Attention was drawn concerning the use of probiotics in immunocompetent patients.	[145]
	*Saccharomyces* spp., *Lactobacillus* spp., *Bifidobacterium* spp., *Bacillus* spp.	Fungemia Endocarditis Abscess Pneumonia, Pleural empyema Septic arthritis *Saccharomyces Lactobacillus Bifidobacterium* *Bacillus*	In a systematic review of adverse reactions to probiotics in the main international databases published by August 2018, a total of 93 patients were analyzed. Fungemia was the most common infectious complications in 37.6% cases. Genus *Saccharomyces* was the most frequent in 50.6% cases, followed by *Lactobacillus*, *Bifidobacterium*, *Bacillus*, *Pedioccocus* and *Escherichia* in 27.9%, 12.8%, 5.4%, 2.2% and 1.1% cases, respectively. Adults over 60 years of age, *Clostridioides difficile* colitis, antibiotic use and *Saccharomyces* infections were associated with overall mortality. HIV infections, immune-suppressive drugs, solid organ transplantation, deep intravenous lines, enteral or parenteral nutrition were not associated with mortality. Administration of probiotics cannot be considered risk-free.	[146]
	*Saccharomyces cerevisiae var. boulardii*	*Clostridioides difficile*-associated diarrhea	A case of fungemia in a patient suffering from *Clostridioides difficile*-associated diarrhea treated with metronidazole and a probiotic containing *S. cerevisiae var. boulardii*. Fluconazole 400 mg/day was started, and the probiotic was stopped. Potential benefit of *S. cerevisiae var. boulardii* should be accurately evaluated, especially in elderly patients.	[147]
**Disseminated infection**	LGG *ATCC 53103*	Disseminated LGG *ATCC 53103* infection Intrauterine growth restriction	A disseminated LGG ATCC 53103 infection was suspected in a 6-day-old newborn with intrauterine growth restriction symptoms, treated empirically with antibiotics and given LGG with the aim of preventing antibiotic-associated gastrointestinal complications.	[148]
**Empyema**	LGG	*Lactobacillus* empyema Immunodeficiency virus-infected lung transplant	A case of *Lactobacillus* empyema in a patient infected with the human immunodeficiency virus who received a cardiothoracic transplant and a probiotic containing LGG.	[149]
**Risk of** **celiac disease autoimmunity**	*Lactobacillus reuteri* and LGG	Celiac Disease Autoimmunity	Aim of the study was to investigate the relationship between probiotic use in dietary supplements or infant formulas up to 1 year of age and the occurrence of celiac disease autoimmunity (CDA) and celiac-like disease among a cohort of 6520 genetically susceptible children. The use of probiotics in the first year of life was detected in 1460 children through the intake of probiotic food supplements, which were associated with a slightly increased risk of CDA, compared to children who did not receive probiotics.	[150]

**Table 4:** Probiotics and respiratory tract infections.

Type of Study	Probiotics	Targets/Types of Respiratory Tract Infections	Results	Reference
Animal study	VSL#3 probiotic: *Bifidobacterium breve*, *Bifidobacterium longum*, *Bifidobacterium infantis*, *Lactobacillus* *acidophilus*, *Lactobacillus plantarum*, *Lactobacillus paracasei*, *Lactobacillus bulgaricus*, *Streptococcus thermophiles*	Biopsies of colon and jejunum tissues, and inguinal or axillary lymph nodes. Cellular and humoral immunity and inflammation in healthy macaques	Daily treatment with the VSL#3 probiotic (PBio) resulted in significantly increased frequencies of B-cells expressing IgA in the colon and lymph node (LN), likely because of significantly increased LN T follicular helper cell frequencies and LN follicles. Increased frequencies of IL-23 + Antigen-presenting cells (APCs) in the colon were found post-PBio treatment, which correlated with LN T follicular helper cells. VSL#3 significantly downmodulated the response of TLR2-, TLR3-, TLR4-, and TLR9-expressing HEK293 cells. Beneficial impact of PBio on mucosal health and the possibility of using probiotics in the context of vaccination or prevention against mucosal infections.	[217]
Systematic review	*Bifidobacterium longum* BB536 *Lactobacillus plantarum* L-137 *L. plantarum* DK 119 *Lactobacillus paracasei* *L. rhamnosus* CRL 1505 *L. reuteri DSM 1793* *L. gasseri* TMC0356 *B. animalis* subsp. *Lactis* BB12	Acute respiratory tract infections (pneumonia, influenza, enterovirus, adenovirus, and respiratory syncytial virus infections) caused by DNA/RNA viruses. COVID-19	Purpose of this review was to summarize existing information on the gut mediated-pulmonary immunity conferred by probiotics. Recent evidence has shown an association between COVID-19 disease and intestinal dysbiosis. Due to the proved close relationship of the gastrointestinal and respiratory tract, the dysfunction of the first may trigger disease in the last. Probiotics could reshape the composition of the intestinal microbiota and consequently, regulate immune responses in the respiratory system. Probiotic strains and their metabolites, such as bacteriocins, have been studied as potential antiviral agents. Due to the high mutational rate of RNA viruses and a major challenge of restricted antibiotic efficacy, probiotic administration would help increase host immunity and, similar to other antiviral studies, could reduce the symptoms of the new coronavirus. Probiotics have become a nutraceutical and promising immunobiotic agent to possibly treat COVID-19 infection following the absence of a vaccine or a proven therapeutic intervention.	[219]
Animal study	Heat-killed *Lactobacillus plantarum* L-137 (HK-LP)	Influenza virus infection	C57BL/6 mice intranasally infected with influenza virus A/FM/1/47 (H1N1, a mouse-adapted strain) were administered orally HK-LP. Survival time was significantly prolonged, an appreciable level of interferon (IFN)-β was detected in the serum, and the viral titers in the lung were significantly lower in mice treated with HK-LP than controls. No IFN-β was detected in controls after influenza infection. HK-LP, a potent IFN-β inducer, could prevent against influenza infection.	[220]
Animal study	*Lactobacillus casei* strain Shirota (LcS)	Upper respiratory influenza virus (IFV) infection	Mice were intranasally administered *Lactobacillus casei* strain Shirota (LcS) and a strong production of interleukin 12, gamma interferon, and tumor necrosis factor alpha was proved in mediastinal lymph node cells, very important in excluding influenza virus (IFV). Titers of virus in the nasal wash of mice inoculated with 200 μg of LcS for three consecutive days (LcS 200 group) before infection were significantly (*p* < 0.01) lower than those of mice not inoculated with LcS (control group) (100.9 ± 0.6 versus 102.1 ± 1.0). The survival rate of the mice in the LcS 200 group was significantly (*p* < 0.05) greater than that of the mice in the control group (69% versus 15%). Decrease in the titer of virus in the upper respiratory tract to 1/10 of the control level was important in preventing death. Intranasal administration of LcS enhances cellular immunity in the respiratory tract and protects against influenza virus infection.	[224]
	*Lactobacillus pentosus* strains, *L. casei* Shirota, *L. plantarum* strains, *L. delbrueckii* subsp. *bulgaricus* OLL1073R1, LGG, *L. gasseri* TMC0356, *Lactococcus lactis* subsp. *cremoris* FC, *L. brevis* KB, *B. breve* YIT4064	Influenza virus infection in mice	Oral or intranasal administration of mentioned probiotics have reduced the infection, virus titer in the lungs or nasal washings, and increased mice survival.	
Systematic review	*L. plantarum* NCIMB 8826 *L. reuteri* F275	Pneumovirus infection in mice	Virus-induced inflammation was suppressed, and the mice were protected against lethal disease.	[225]
	*L. rhamnosus* CRL1505 *L. rhamnosus* CRL1506	Respiratory syncytial virus infection	Nasally administered probiotics differentially modulated immune responses and induced protection against respiratory syncytial virus infection.	
In vivo and in vitro animal study on BALB/cCrSlc mice	*Lactobacillus gasseri* SBT2055 (LG2055)	Antiviral activity against respiratory syncytial virus (RSV) on HEp-2 human laryngeal epithelial cells and MLE12 mouse lung epithelial cells. Pro-inflammatory cytokines TNF-α, CCL2, IL-1β, and IL-6 in lung tissue. Proteomic analysis of a total of 1120 proteins	LG2055 inhibited RSV replication in vitro and in vivo and suppressed the inflammatory response in the lungs of mice. LG2055 enhanced IFN-β and IFN-γ expression at the gene level in the lungs of mice, decreased the expression of SRCAP, one of the most strongly LG2055-down-regulated protein, and inhibited the RSV replication. LG2055 is a promising probiotic useful for preventing RSV infection and relieving the associated symptoms.	[302]
Clinical study on elderly (randomized and controlled)	*Lactobacillus* *delbrueckii* subsp. *bulgaricus* OLL1073R-1. 100 g of 1073R-1-yogurt for 12 weeks. Control participants consumed yogurt fermented with a different *Lactobacillus* strain (control yogurt).	Influenza A virus subtype H3N2-bound	Consumption of 1073R-1-yogurt affected influenza A virus subtype H3N2-bound IgA levels in saliva. In addition, saliva flow rate and total IgA levels increased in response to the yogurt intake period in both the 1073R-1 and control yogurt group. Continuous daily ingestion of 1073R-1-yogurt may help prevent infection with influenza A virus subtype H3N2 in elderly with weakened immunity.	[303]
Clinical trial (double-blind randomized, placebo-controlled)	*L. plantarum* DR7, isolated from bovine milk; 9 log CFU/day for 12 weeks	Health conditions via monthly questionnaires, and cytokine concentrations, peroxidation, oxidative stress, and gene expression in T-cells and natural killer (NK) cells from blood samples were assessed for upper respiratory tract infections (URTI), during the 12-wk intervention period	DR7 reduced the duration of nasal symptoms and the frequency of URTI, compared to placebo. DR7 suppressed plasma pro-inflammatory cytokines (IFN-γ, TNF-α) and increased anti-inflammatory cytokines (IL-4, IL-10); it reduced plasma peroxidation and oxidative stress levels compared to placebo group. A higher expression of plasma CD44 and CD117, and a lower expression of plasma CD4 and CD8 compared with the placebo, indicating less T-cell activation. Enhanced presence of non-resting and mature NK cells compared to placebo. DR7 treatment alleviated the symptoms of URTI by improving inflammatory parameters and enhancing immunomodulatory properties and could be suitable for food or health applications.	[304]
Prospective clinical trial (double-blind randomized, placebo-controlled)	Daily probiotic drink (150 mL) that contained *L. paracasei* at 3 × 10^7^ CFU/mL, *L. casei* 431 at 3 × 10^7^ CFU/mL, and *L. fermentum* PCC at 3 × 10^6^ CFU/mL; or *placebo* drink administered after lunch, for 12 weeks.	136 adults diagnosed with common cold or influenza-like respiratory illness (collectively upper respiratory infections (URI)) at least four times in the previous year were enrolled. Blood and fecal samples were collected at two time points: at baseline, and at 12 weeks. Subject compliance was followed by daily questionnaires	Probiotics significantly reduced the incidence of URIs and influenza-like symptoms with an oral temperature higher than 38 °C compared to the placebo group. The probiotic group had a significantly higher level of IFN-γ in serum and sIgA in the intestine compared to the placebo group and compared to the results of the initial tests. In contrast, there were no significant differences in serum with respect to IL-4, IL-10, IgA, IgG or IgM between probiotics and placebo groups. Probiotics have been safe and effective in combating the common cold and flu-like respiratory infections by stimulating the immune system.	[305]
Clinical study on 205 volunteers aged ≥45 years (double-blind randomized, placebo-controlled)	300 mL/day of yogurt supplemented with *Lactobacillus paracasei* N1115, 3.6 × 10^7^ CFU/mL for 12 weeks. Control group, normal diet without any probiotic	Incidence of URTI, and changes in serum protein, immunoglobulins, and the profiles of the T-lymphocyte subsets (total T-cells [CD3+], T-helper cells [CD4+], and T-cytotoxic-suppressor cells [CD8+])	The risk of URTI in the intervention group was assessed as 55% of that in the control group. The change in the percentage of CD3+ cells in the intervention group was significantly higher than in the control group, but no significant differences were observed in the total levels of protein, albumin, globulin and prealbumin in both groups. Therefore, N1115 may reduce the risk of acute URTI in the elderly. Improving natural T-cell-mediated immune defense could be one of the important mechanisms underlying probiotics to express their anti-infective effects.	[306]

**Table 5:** Effects of photobiomodulation on microbiome and disease management.

Type of Study	PBM Parameters and Protocol	Performed Analysis	PBM Effects	Reference
Experimental study with red laser on *L. casei* NRRL-B-1922	Red laser 632.7 nm, 40 mW; 3, 6, 12 J/cm^2^; exposure time 10, 20, 40 min, respectively.	PBM (red laser exposure) applied to *L. casei* NRRL-B-1922 before the fermentation of skim milk.	Exposure of *L. casei* NRRL-B-1922 to the dose of 12 J/cm^2^ before skimmed milk fermentation exhibited a significant improvement of the anti-oxidant capacity, β-galactosidase, antimicrobial, and proteolytic activities. It decreased the cholesterol and lactose levels of fermented skimmed milk, enhancing the fermentation process of skimmed milk prepared with *L. casei* NRRL-B-1922. It opens the perspective of red laser photobiomodulation of probiotic bacteria during the fermentation process of skimmed milk to improve the quality of fermented milk on an industrial scale, with significant economic benefits, too.	[307]
Animal study on BALB/c mice	Abdomen irradiated with red (660 nm), output power 75 mW, power density 93.75 mW/cm^2^; or, infrared (808 nm), output power = 83 mW, power density = 103.75 mW/cm^2^, either as single or multiple doses, over a two-week period. Spot size = 0.8 cm^2^ for both lasers, pulse frequency of 250 Hz. Each mouse received a total energy density of 10 J/cm^2^. Sham treatments were identical.	Genomic DNA extracted from fecal pellets was pyrosequenced for the 16S rRNA gene.	*Allobaculum* bacterium, associated with a healthy microbiome, significantly increased (*p* < 0.001) after infrared (but not red light) PBM by day 14. It is the first experiment proving that PBM can alter microbiome diversity in healthy mice and increase numbers of *Allobaculum*. If confirmed in humans, it opens avenues for PBMT to be applied as an auxiliary treatment in obesity, cardiovascular and neurodegenerative diseases, as well as other disorders.	[266]
Animal study on C57BL/6 N mice	PBM was performed on the abdomen of the mice at the wavelengths of 630 nm, 730 nm, and 850 nm. Irradiation time was 1000 s (16 min and 40 s), the power density was 10 mW/cm^2^, and the energy density was 100 J/cm^2^, once a day, 5 times a week, for 8 weeks.	Gut flora-targeted PBM (gf-targeted PBM) on Alzheimer’s disease (AD) animal model. Expression levels of 509 proteins, which involved the pathways of hormone synthesis, phagocytosis, and metabolism. The 16 s rRNA gene sequencing of fecal contents.	Gf-targeted PBM reversed the imbalance of intestinal flora and improved learning ability, amyloid plaque deposition, tau phosphorylation, and microglia inflammation of Aß-induced AD mice. Many proteins in the hippocampus responded to gf-targeted PBM, with mitochondrial respiratory chain complex enzymes as a possible key intermediate target. PBM significantly altered the diversity and abundance of intestinal flora, reversing the typical increase of *Helicobacter* and uncultured *Bacteroidales*, and the decreasing the *Rikenella* seen in AD mice. Gf-targeted PBM has the potential to be a noninvasive microflora regulation method for Alzheimer’s disease patients. Future studies will confirm the effect of gf-targeted PBM on the brain-gut axis, promoting PBM as a potential prevention and treatment method for AD.	[308]
Animal study on Sprague-Dawley (SD) rats	PBM with IR (830 nm, 100 mW/cm^2^) supplementary light irradiation was carried out from 14:00 to 14:30 every day, for three months. Illuminance in the feeding box was 1000 lx.	Concentration of bone metabolism markers, including 1,25-dihydroxyvitamin D3 (1,25-(OH)2-D3), bone-specific alkaline phosphatase (BALP), and tartrate-resistant acid phosphatase (TRACP), were detected from blood samples in four study groups. Whole body, femur and tibia of the rats were scanned with a dual-energy X-ray bone densitometer. Bacterial genomic DNA was extracted from the frozen stool samples with a DNA extraction kit. The V3-V4 region of the 16S rRNA (341F-805R), F: GATCCTACGGGAGGCAGCA; R: GCTTACCGCGGCTGCTGGC) was studied. An open-source R package, Tax4Fun, was first used to analyze the enrichment of functional genes of the microbiome of each group.	Analysis of the structure and function of gut microbiota in the rats after PBM infrared supplementation significantly reduced the abundance of Saccharibacteria and increased the abundance of Clostridiaceae 1 and Erysipelotrichaceae bacteria. Results proved that changes in the gut microbiome correlate well with bone mass and bone metabolism. Infrared supplementation can have a positive effect on rat bone metabolism by affecting gut microbiota. These findings could be used in the future design of healthy lighting environments that prevent or possibly ameliorate osteoporosis.	[309]
Animal study on C57BL/6 mice	PBM with laser at 660 nm and radiant exposure of 10 J/cm^2^, was applied six hours after intratracheal inflammation produced with instillation of lipopolysaccharide (LPS) (5 mg/kg) or phosphate buffer saline (PBS).	Inflammatory cells in perivascular and alveolar spaces, and inflammatory mediator secretion.	Increased expression and secretion of cytokines (TNF-α, IL-1β, IL-6,) and chemokine (MCP-1). PBM induced a significant decrease in both inflammatory cell influx and inflammatory mediator secretion. PBM did not affect the mechanical properties of the lungs, nor the strength of the tissue, nor the elasticity. PBM reduced the inflammatory reaction in the lungs exposed to LPS without affecting lung function and recovery.	[288]
Animal study on BALB/c mice	PBM (830 nm laser, 9 J/cm^2^, 35 mW, 80s per point, 3 points per application) was applied in direct contact with skin, 1 h after LPS administration. Mice were distributed in control (n = 6; PBS), ARDS IT (n = 7; LPS orotracheally 10 μg/mouse), ARDS IP (n = 7; LPS intra-peritoneally 100 μg/mouse), ARDS IT + Laser (n = 9; LPS intra-tracheally 10 μg/mouse), ARDS IP + Laser (n = 9; LPS intra-peritoneally 100 μg/mouse).	LPS-induced pulmonary and extrapulmonary acute respiratory distress syndrome (ARDS). 24 h after last LPS administration, mice were studied for pulmonary inflammation by total and differential cell count in bronchoalveolar lavage (BAL), cytokines (IL-1beta, IL-6, KC and TNF-alpha) levels in BAL fluid and by quantitative analysis of neutrophils number in the lung parenchyma.	PBM significantly reduced pulmonary and extrapulmonary inflammation in LPS-induced ARDS, reduced number of total cells and neutrophils in BAL, reduced levels of IL-1beta, IL-6, KC and TNF-alpha in BAL fluid and in serum, as well as the number of neutrophils in lung parenchyma. PBM was efficient in reducing pulmonary inflammation in both pulmonary and extrapulmonary model of LPS-induced ARDS.	[289]
In vitro study of dermal fibroblast cell line (HFF-1) with premature senescence H_2_O_2_-induced	PBMT: 660 nm, energy density = 3, 4, 5, 6, and 8 J/cm^2^; power density = 35 mW; time = 10 s, 14 s, 16 s, 20 s, and 28 s. Beam area = 0.035 cm^2^, beam diameter = 0.21 cm^2^, frequency 16 Hz, pulsed. Number of points 8. Area of the laser application 9.6 cm^2^. Contact/No contact—distance of 35 mm. Cellular mortality, proliferation, and the levels of oxidative, inflammatory cytokines, apoptotic markers, and of two growth signaling molecules (FGF-1 and KGF) were compared among treatments.	Protein quantification of the following markers: DNA 8-deoxyguanosine and cytokines involved in inflammatory response interleukin IL-1β, IL-6, IL-10, tumoral necrosis factor alfa (TNF-α), and interferon-gamma (IFN-γ). Caspase-1, caspase-3, and caspase-8 activities were determined by assay kits, fluorometric.	Interaction between H_2_O_2_ at 50 μM and PBM at 4 J (best dose) showed partially reversion of the higher levels of DNA oxidation, CASP 3, CASP 8, IL-1B, IL-6, and IFN-γ induced by H_2_O_2_ exposure. PBM also trigger increase of IL-10 anti-inflammatory cytokine, FGF-1 and KGF levels. PBM on the fibroblast without injury was relative safe and harmless, given its cytogenotoxic potential, oxy-inflammatory, and proliferative effects. However, in the injured H_2_O_2_ fibroblast, PBM had significant protection and proliferative effect, partially or totally reversing the negative effects triggered by H_2_O_2_. At certain dose ranges, PBM may trigger anti-aging properties.	[291]
In vitro model of human keratinocytes cell line (HaCaT) infected with Herpes Simplex Virus Type-1 (HSV-1)	HSV-1 were irradiated using a diode laser device (class IV) with the following two protocols: 445 nm, 0.3 W/cm^2^, 60 J/cm^2^, CW, or 445 nm, 0.15 W/cm^2^, 30 J/cm^2^, 5 Hz.	After 30 min the virus irradiated and not irradiated was transferred to a HaCaT cells culture and then, after another 24 h HSV-1 quantification was performed on the cell supernatants. Five experimental settings were used and the increase in cell vitality and the decrease in HSV-1 viral load in supernatants of previously irradiated virus-infected cells were measured comparatively with non-irradiated virus-infected cells.	Experimental results proved that the blue laser has antiviral activity against HSV-1, and it is more effective against virus irradiated alone, suggesting that PBMT inactivates the virus prior to cell entry. In contrast, when the virus is already inside the cells, the effect of PBMT is less evident and does not increase cells’ resistance to infection. Blue PBM had a direct inhibitory effect on the virus itself. Further studies are necessary to determine how blue PBM exerts its antiviral effect, the aim being to move from an in vitro to a clinical setting, thus promoting its use on HSV-1 infected patients.	[310]
In vitro cellular model of hidradenitis suppurativa (HS) on human keratinocyte cell line (HaCaT)	Two irradiation protocols with near-infrared (NIR) and Blue PBM: 970 nm, 0.3 W/cm^2^, 20 J/cm^2^, continuous wave (CW) and 445 nm, 0.2 W/cm^2^, 10 J/cm^2^, CW, using fluency at 10–30–50 J/cm^2^.	Effect of PBM on IL1B gene (encoding for interleukin-1β [IL-1β]) expression in immortalized human keratinocyte cell line using a wild-type line and a knockout cell model mimicking genetic-driven Hidradenitis Suppurativa (HS).	Based on the hypothesis that increased production of pro-inflammatory cytokines would promote a dysbiosis of resident skin microbes and so, the perpetuation of skin inflammation in HS, it was shown that PBM decreased IL1B gene expression, which could block the up-mentioned vicious mechanism. PBM could be a useful tool in the management of skin lesions in patients with HS.	[311]
In vitro model of SARS-CoV-2 infection	PBMT using LEDs at 450 nm with 12.5 J/cm^2^; 454 nm with 10 J/cm^2^; 470 nm with 20 J/cm^2^; irradiance of 40 mW/cm^2^, continuous waves.	Experiments were performed on Vero E6 epithelial normal cell line derived from the kidney of *Cercopithecus aethiops* (ATCC CRL-1586), with three experimental settings: SARS-CoV-2 was irradiated and then transferred to cells; already infected cells were irradiated; cells were irradiated prior to infection.	Results may support the possible exploitation of blue light to meet the challenges of SARS-CoV-2, as blue wavelengths have stopped SARS-CoV-2 replication. The antiviral activity of PBMT against SARS-CoV-2 on human cell lines is intended to propose translatability for this new approach to support individuals affected by COVID-19, also considering that PBMT is largely safe, with no side effects, and well tolerated by patients.	[312]
Systematic review	PBM using red to infrared light (λ = 600–1070 nm) has been analyzed in several pre-clinical models of Alzheimer’s and Parkinson’s disease, as an emerging putative neuroprotective therapy.	Tissue stressed by hypoxia, toxic insult, genetic mutation and mitochondrial dysfunction.	Analysis proved important reductions in β-amyloid plaques, neurofibrillary tangles of hyperphosphorylated tau protein, inflammation and oxidative stress, together with increased ATP levels and improved overall mitochondrial function as follows: increase (↑) Cell survival (striatal and cortical cells), ↑T-Helper + cells, ↑ATP content, ↑Complex IV-dependent respiration, decrease (↓) Oxidative stress, ↓Inflammation, ↑Mitochondrial function, ↑Heat shock proteins, ↓Amyloid aggregates, ↓Hyperphosphorylated tau. In addition, PBM reduced the characteristic cognitive deficits in transgenic mouse models.	[268]
Systematic review	A literature search was conducted for published reports on the effect of PBM [visible or near-infrared (NIR)] on the microbiome, red (630–680 nm) or in the NIR region (780–940 nm) and (980 and 1064 nm). Power densities: 10–100 mW/cm^2^, energy densities in the region of 4–50 J/cm^2^.	Subcellular, cellular (neurons, epithelial cells, keratinocytes, fibroblasts etc.) and tissue levels. Organ level: brain (oscillation patterns), gut etc. Microbiome.	The following conclusions can be drawn: A. Light can affect the microbiome indirectly through the daily circadian rhythm. B. Light has an indirect effect on the microbiome through vitamin D, produced by the action of sunlight on keratinocytes. C. PBM effects on cytochrome c oxidase (CCO): ↑CCO, ↑Mitochondrial membrane potential, ↑ATP production, brief burst of reactive oxygen species, ↑nitric oxide, ↑cyclic AMP, ↑movements in intracellular calcium, ↑transcription factors, ↑expression of a multitude of gene, ↑structural proteins, ↑enzymes, ↑cell division, ↑cell migration. D. PBM effects at cellular and tissue level: ↑nonvisual phototransduction cascades involving opsins. Blue (415 nm) and green (540 nm) light absorbed by opsins, trigger opening of transient receptor potential (TRP) calcium ion channels. E. PBM on inflammatory pathways: ↓pro-inflammatory cytokines (IL-6, TNF-α, IFN-γ). F. PBM on immune system: ↑circulating immune cells (mast cells, macrophages, etc.), transduce protective signals from distal tissues to sites of injury (brain, heart, or gut). E. PBM on microbiome: ↑*Akkermansia muciniphila*, ↑*Bifidobacterium* spp., ↑*Faecalibacterium* spp., and ↓Firmicutes/Bacteroidetes ratio.	[270]
Randomized clinical trial with COVID-19 pneumonia	Two laser sources (808 nm and 905 nm), working simultaneously and synchronously as follows: 1. Three GaAlAs laser diodes, 808 nm, peak power of 1 W, average power 500 mW each diode, in total 1.5 W, power density 75 mW/cm^2^, 1500 Hz, duty cycle of 50%, pulse duration of 330 µs, spot size of 19.6 cm^2^. 2. Three superpulsed GaAs laser diodes, 905 nm, peak power 75 W, average power 203 mW each diode, in total 610 mW, power density 31 mW/cm^2^, 1500 Hz (train pulses 90 kHz modulated at 1 Hz ÷ 2 kHz), pulse duration of 100 ns, spot size of 19.6 cm^2^. Each lung was scanned for 14 min, from apex to base, over an area of 250 cm^2^ of the posterior thorax, resulting in 28 min of PBMT with a dosage of 7.18 J/cm^2^ and a total energy of 3590 J.	PBMT group received standard medical care plus adjunctive PBMT, four daily sessions of near-infrared light treatment targeting the lung tissue. Control group received only standard medical care. Patient outcomes were measured via blood work, chest x-rays, pulse oximetry and validated scoring tools for pneumonia.	PBMT-treated patients showed rapid recovery, did not require ICU admission or mechanical ventilation, and reported no long-term sequelae at 5 months after treatment. In the control group, 60% of patients were admitted to the ICU for mechanical ventilation. The control group had an overall mortality of 40%. At a 5-month follow-up, 40% of the control group experienced long-term sequelae. PBMT is a safe and effective potential treatment for COVID-19 pneumonia and improves clinical status in COVID-19 pneumonia.	[313]
PDT clinical trial with COVID-19 in the early stage of infection	Laser light watch with 4 red laser diodes (658 nm), 2 blue (447 nm), 2 green (532 nm) and 2 yellow (589 nm) LEDs for systemic treatment of blood via the wrist arteries for 60 min; one nose treatment applicator with 1 blue LED (447 nm) and 1 UVA LED (375 nm), 10 min each nostril with blue and UVA light (sides switched after 10 min); one mouth treatment applicator with 14 blue LEDs (447 nm) and 14 UVA LEDs (375 nm) for 20 min inside the mouth and throat. As photosensitizer for photodynamic therapy (PDT): 2 capsules Riboflavin-5phosphate 100 mg/each treatment, as follows: one capsule for systemic application taken 1 h before starting the PDT, and the second one (100 mg) dissolved into a glass of 200 mL water (for local application in nose, mouth and throat).	Two groups with 20 patients each: one group receiving PDT and daily testing, and a control group receiving conventional care plus testing. All patients in both groups had positive Covid-19 test results at the beginning of the study being in an early infection stage with mild symptoms such as fever, dry cough, headache, hard breathing, fatigue etc. QPCR tests with CT-viral load were performed on day 1, 2, 3, 4, 5 and 7 in the PDT group, and on day 1, 3, 5 and 7 in the control group.	All 20 patients in the PDT group showed significant improvement in clinical symptoms and viral load assessment within the 5 days of PDT. 14 out of 20 patients had a negative QPCR test after 5 days of PDT, while the other 6 patients also showed significantly reduced viral load. All 20 patients in the control group were tested 3 times within 5 days and no significant improvement could be seen clinically or in the viral load assessment.	[314]

## Data Availability

The references used to support the findings of this review article are available from the first and the corresponding authors upon request.
